# Atomically Resolved Electrically Active Intragrain Interfaces in Perovskite Semiconductors

**DOI:** 10.1021/jacs.1c12235

**Published:** 2022-01-21

**Authors:** Songhua Cai, Jun Dai, Zhipeng Shao, Mathias Uller Rothmann, Yinglu Jia, Caiyun Gao, Mingwei Hao, Shuping Pang, Peng Wang, Shu Ping Lau, Kai Zhu, Joseph J. Berry, Laura M. Herz, Xiao Cheng Zeng, Yuanyuan Zhou

**Affiliations:** † Department of Applied Physics, The Hong Kong Polytechnic University, Hong Kong SAR 999077, People’s Republic of China; ‡ Department of Chemistry, University of Nebraska-Lincoln, Lincoln, Nebraska 68588, United States; § Qingdao Institute of Bioenergy & Bioprocess Technology, Chinese Academy of Sciences, Qingdao, Shandong 458500, People’s Republic of China; ∥ Clarendon Laboratory, Department of Physics, University of Oxford, Oxford OX1 3PU, United Kingdom; ⊥ Department of Physics, Hong Kong Baptist University, Kowloon, Hong Kong SAR 999077, People’s Republic of China; # College of Engineering and Applied Sciences and Collaborative Innovation Center of Advanced Microstructures, Nanjing University, Nanjing 210093, People’s Republic of China; ∇ Department of Physics, University of Warwick, Coventry CV4 7AL, United Kingdom; △ Chemistry and Nanoscience Center, National Renewable Energy Laboratory, Golden, Colorado 80401, United States; ● Smart Society Laboratory, Hong Kong Baptist University, Kowloon, Hong Kong SAR 999077, China; □ Material Science Center, National Renewable Energy Laboratory, Golden, Colorado 80401, United States; ■ Renewable and Sustainable Energy Institute and the Department of Physics, University of Colorado Boulder, Boulder, Colorado 80309, United States

## Abstract

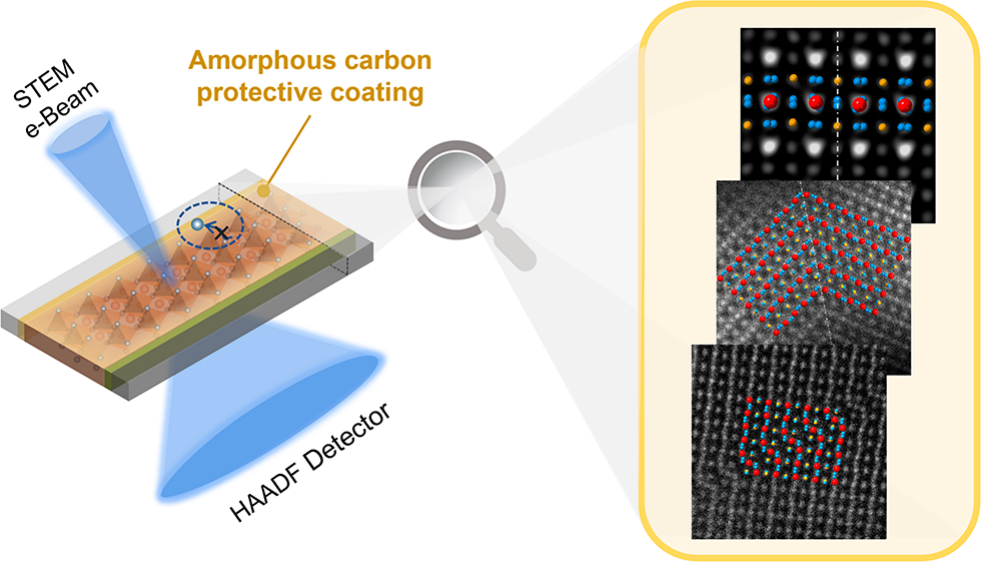

Deciphering the atomic and electronic structures of interfaces is key to developing state-of-the-art perovskite semiconductors. However, conventional characterization techniques have limited previous studies mainly to grain-boundary interfaces, whereas the intragrain-interface microstructures and their electronic properties have been much less revealed. Herein using scanning transmission electron microscopy, we resolved the atomic-scale structural information on three prototypical intragrain interfaces, unraveling intriguing features clearly different from those from previous observations based on standalone films or nanomaterial samples. These intragrain interfaces include composition boundaries formed by heterogeneous ion distribution, stacking faults resulted from wrongly stacked crystal planes, and symmetrical twinning boundaries. The atomic-scale imaging of these intragrain interfaces enables us to build unequivocal models for the *ab initio* calculation of electronic properties. Our results suggest that these structure interfaces are generally electronically benign, whereas their dynamic interaction with point defects can still evoke detrimental effects. This work paves the way toward a more complete fundamental understanding of the microscopic structure–property–performance relationship in metal halide perovskites.

## Introduction

Metal halide perovskites (MHPs) are an emerging class of semiconductors with the chemical formula of ABX_3_, where A is a monovalent organic or metal cation, B is a divalent metal cation, and X is a halide ion.^1,2^ These semiconductors can be easily processed into thin films at low temperatures using various methods, and their compositions and properties are highly tunable, demonstrating promising applications in various optoelectronics.^1^ Especially, perovskite-based solar cells (PSCs) have experienced a swift increase in power conversion efficiencies (PCEs) in the past few years.^3^ This has been enabled by a great number of fundamental research works that involve revealing and tailoring internal interface structures in MHP thin films.^4−13^ Previous studies concerning interfaces have mainly relied on conventional characterizations, such as optical spectroscopy, scanning electron microscopy, and scanning probe microscopy, with spatial resolutions limited to only micro-/nanometer scales.^1,14−18^ In this regard, those apparent grain boundaries (GBs) are frequently the only internal interfaces visible in the studies, whereas a considerable density of intragrain interfaces (IGIs) has recently been confirmed to exist in MHPs.^19^ Omitting such IGIs potentially causes a misinterpretation of the role of GBs in MHP properties and PSC performance, and this can be one possible cause for the discrepancy in the current understanding of the microstructure–property–performance relationship. Nevertheless, these IGIs do not exhibit noticeable morphological features such as domain boundaries and they are frequently buried underneath the top surfaces, preventing direct observation and characterization using conventional methods. Furthermore, the structural details revealed are much less than sufficient for gaining critical insights into the atomistic landscape of all interfaces, which are essential to an in-depth mechanistic understanding of the properties.^12,20^

Transmission electron microscopy (TEM) is one of the most powerful tools for structural characterizations with a high spatial resolution up to the atomic or even subatomic scale.^13,21,22^ TEM has been applied to MHP research,^23,24^ but the low radiolysis tolerance of MHPs upon incident high-energy electron beams renders it highly challenging to achieve high-quality imaging.^13,22^ As a result, the quality and reliability of reported imaging results are frequently questioned. Recent advances in TEM characterization are significant, demonstrating its capability in revealing atomic-scale structures of standalone MHP films or nanomaterial samples.^13,25−29^ However, these observations are mostly based on plan views, for which acquired atomic structural information on the internal interfaces may not be directly related to the device functions. This is because PSCs are usually vertical devices where cross-sectional interface microstructures are considered most crucial to the performance. However, the ultrathin nature of cross-section specimens, prepared by a focused-ion-beam (FIB) nanofabrication, further adds to the technical difficulty of TEM characterization.^30^ In this study, we develop a simple yet reliable TEM approach to image MHPs in high-performance PSC devices, which unambiguously reveals the atomic-scale information on three prototypical intragrain interfaces. This enables us to construct accurate, highly correlated theoretical models to elucidate the electronic behaviors of all these intragrain interfaces, exposing their statically benign yet dynamically detrimental role on the potential device performance.

## Results

### TEM Sample Selection and Characterization Development

We chose to study formamidinium–cesium (FA-Cs) mixed-cation MHPs, a highly promising system that can simultaneously deliver both high efficiency and long-term stability in single cells and modules.^31−36^ We prepared FA_1–*x*_Cs_*x*_PbI_3_ thin films across the full range from *x* = 0 to *x* = 1 (Figure S1) using a Cs_4_PbBr_6_-mediated method previously reported.^37^ We then fabricated PSCs by sandwiching the MHP absorber layer with a SnO_*x*_/FTO electron-extracting layer and a Spiro-OMeTAD/Au hole-extracting layer. Details of the MHP synthesis and PSC fabrication are included in the Experimental Section. While with processing optimization all of the FA_1–*x*_Cs_*x*_PbI_3_ devices in the range of *x* = 0.15 to *x* = 0.85 can show high PCEs beyond 17% with good reproducibility in general (Figure S2), the champion cell in this work shows a PCE of 21.4% when the FA_1–*x*_Cs_*x*_PbI_3_ composition is optimized to *x* = 0.5. The extracted parameters from the current density–voltage (*J–V*) scan (Figure S3) show a short-circuit current density of 22.7 mA cm^–2^, an open-circuit voltage of 1.16 V, and a filling factor of 81.0%. The stabilized PCE near the maximum power point is 21% (Figure S4). This PSC also exhibits a PCE retention of more than 95% after 2,160 h storage in a nitrogen-filled glovebox (Figure S5). These device parameters are comparable to those for state of the art methylammonium-free PSCs reported in the literature,^33−35^ and thus, we consider our FA-Cs PSCs prototypical for fundamental studies.

**Figure 1:**
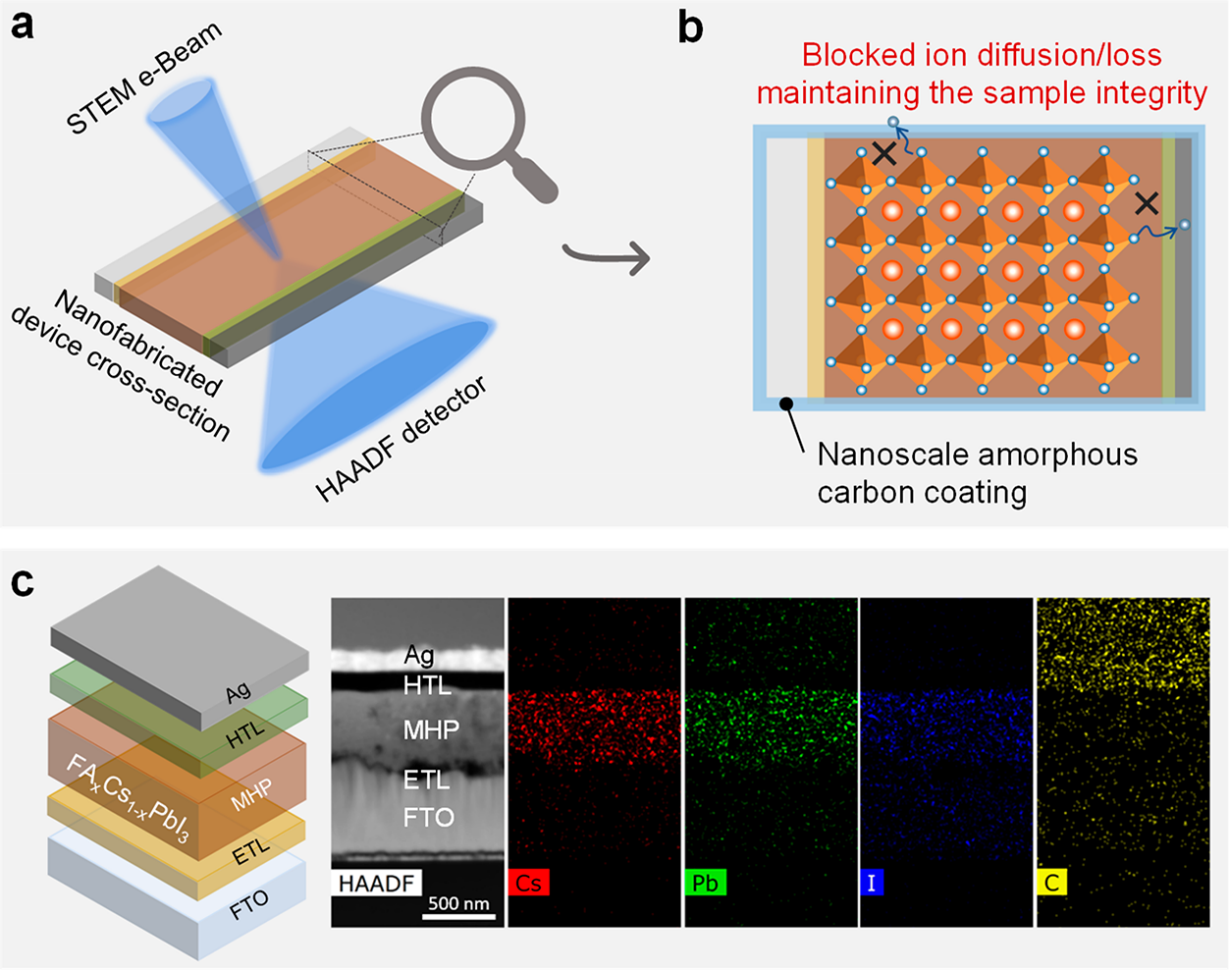
Device sample structure of a typical FA-Cs PSC: (a) schematic illustration of the tailored STEM-HAADF imaging for reliably characterization MHPs in PSCs while the sample integrity is maintained; (b) proposed mechanisms illustrating that the carbon layer retards the evaporation and migration of ions (illustrated by ×) that underpin the sample stability; (c) schematic illustration of the FA-Cs PSC device configuration and corresponding EDS mapping of the device cross-section sample specimen nanofabricated using a focused ion beam.

We then chose scanning transmission electron microscopy (STEM) to image the detailed structures of FA-Cs MHP thin films in PSCs. STEM is an advanced imaging mode of TEM that can enable sub-angstrom spatial resolutions with an aberration correction.^38^ Thus, it directly probes the accurate real-space positions of atoms in samples, and the results from this are more straightforward for structural interpretation than those from conventional TEM imaging. Although STEM has been widely used for characterizing functional materials, including oxide perovskites,^21,39^ it has rarely been applied to MHPs until recently,^13,25,40^ which is due to the relatively high dose rate of the focused electron probe common to STEM. Specialized STEM approaches with lower dose rates and higher detector sensitivities may mitigate these issues, but such facilities are rarely accessible. Therefore, it is vital to develop methods to make it feasible for standard STEM to be used for MHP characterization. Instead of instrumental development, a viable direction is to preserve the sample integrity via additional protection without obviously compressing the imaging quality. Along these lines, after the preparation of device cross-section specimens, we deposited a thin conformal coating of amorphous carbon about 10 nm thick (Figure 1a,b and Figure S6). Surprisingly, we found that this simple step is very effective in protecting MHPs from any damage under a reasonably prolonged exposure to the STEM electron probe. As shown in Figure S7, when the carbon coating is not applied, the focused electron probe can easily trigger the precipitation, which may be composed of Pb, causing a dramatic degradation of the thin-film structures, similarly to the case in previous studies.^41^ However, once the carbon coating is deposited, the microstructures of the entire device are fully retained during our imaging process. We were thus able to perform energy disperse spectroscopy (EDS) mapping for the cross-section specimen, revealing the elemental distribution that delineates each device layer (Figure 1c). As can be seen, the distributions of Cs, Pb, and I elements are all uniform within the FA-Cs MHP thin film at the micrometer scale. As schematically illustrated in Figure 1b, the sample-protecting function of the deposited coating is attributed to the ion-blocking nature of amorphous carbon that impedes the evaporation and migration of ions and thus helps prevents the MHP structure from collapsing. In addition, several other factors involved in our STEM approach may contribute to the minimization of ionization damage, which include enhancing the specimen conductivity due to carbon coating, thinning the FIBed cross-section specimen to below 50 nm, and applying a high electron accelerating voltage (300 kV).^42^ All these enable maintaining the structure of device cross-section specimen even under the scanning electron probe with beam currents up to several pA, which is much higher than that in recent STEM characterization of MHPs by Rothmann et al.^13^ Furthermore, we employed high angle annular dark-field (HAADF), rather than low angle annular dark-field (LAADF) mode for STEM imaging to acquire scattered electrons at a higher space angle. The higher electron dose rate enables the acquisition of high-contrast atomic-scale STEM-HAADF images with an acceptable signal to noise ratio (SNR), although LAADF is potentially superior in detecting a larger fraction of scattered electrons.^22^ Importantly, STEM-HAADF imaging is sufficiently sensitive to atomic number (*Z*) variations, allowing us to distinguish different atomic columns and reveal more detailed information.

To confirm the fidelity of our STEM characterization in revealing the atomic structure of PSCs, we performed a series of continuous electron probe scanning tests that prove the structural robustness of carbon-coated PSC specimens (Figures S8 and S9). As shown in Figure S8, the MHP structures adjacent to an existing PbI_2_ cluster show no obvious change rather than collapsing to PbI_2_ even after a continuous electron probe scanning for over 6 min.^43^ Another test further demonstrated the direct decomposition pathway of the carbon-layer-protected cross-sectional PSC sample under a longer time of electron probe scanning (Figure S9). With over 23 min of continuous electron probe scanning, the MHP only exhibits a slow degradation directly to an amorphous structure. During this degradation process, the structure of the remaining MHP part remains unchanged without phase transformation to nonperovskites or decomposition to PbI_2_. Therefore, the carbon protection and low-dose STEM imaging conditions mitigate the potential influence of electron beams on the MHP structures. Thus, we can conclude that our STEM imaging truly reflects the original atomic structures of the MHP samples. On the basis of this advanced method, we were then able to clearly identify three types of intragrain interfaces in the MHP devices and resolve their atomic structures with a spatial resolution of 1.25 Å at a 300 kV accelerating voltage in STEM.

### Atomic Microstructures of Intragrain Interfaces

**Figure 2:**
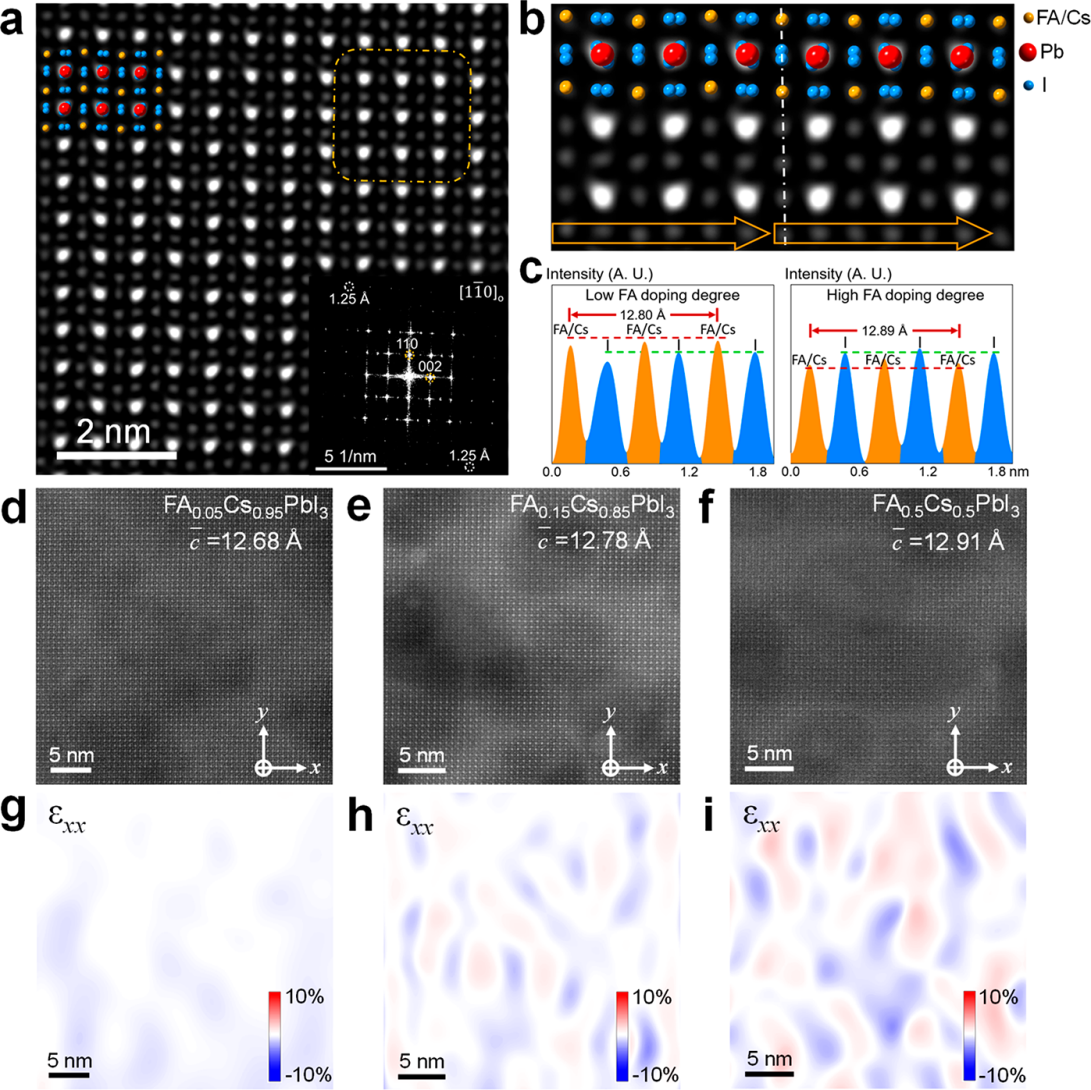
Atomic-scale structures of intragrain composition-boundary interfaces. (a) Atomic-scale Bragg filtered STEM-HAADF image of an orthorhombic FA_0.15_Cs_0.85_PbI_3_ grain from [11̅0] projection direction. The inset gives the corresponding FFT pattern, revealing a spatial resolution of 1.25 Å. (b) Fine atomic structure of the FA-rich domain (right part, as marked by the yellow square in (a)) and intragrain composition-boundary interface, indicating a nearly straight shape of adjacent FA/Cs and I columns in the FA-rich MHP domain (right part) but a zigzag shape in the Cs-rich MHP domain (left part). (c) Line profiles of the row of FA/Cs and I columns signal intensity marked by orange arrows in (b), indicating a higher contrast of FA/Cs columns in comparison to I columns in the Cs-rich domain but a slightly higher contrast of I columns in comparison to FA/Cs columns in FA-rich domain. The measured spacing across three neighboring FA/Cs columns is 12.80 Å in the Cs-rich MHP domain and 12.89 Å in the FA-rich cluster. (d–f) Atomic-resolution unfiltered STEM-HAADF images of FA_1–*x*_Cs_*x*_PbI_3_ samples with different levels of FA incorporation: *x* = 0.95, 0.85, 0.5, respectively. The average lattice spacing *c* increases with a higher level of FA incorporation. (g–i) Corresponding in-plane strain *ε*_*xx*_ distributions of (d–f) generated by a GPA analysis.

On the basis of the high beam tolerance of the sample, we finely tuned the zone axis of one representative MHP grain before imaging. Figure 2a is a typical STEM-HAADF image of the FA-Cs (FA_0.15_Cs_0.85_PbI_3_) MHP grain interior (Bragg filtered as demonstrated in Figure S10), revealing a clear and fine atomic structure. The fast Fourier transform (FFT) pattern (Figure 2a, inset) indicates that the spatial resolution of our STEM-HAADF image is as high as 1.25 Å, comparable to previous cryo-TEM results.^28^ This MHP grain is determined as an orthorhombic phase (space group *Pnma*) and is projected along the [11̅0] direction. The type of each atomic column can be identified from its corresponding characteristic contrast (*Z* contrast), similarly to STEM-HAADF observations of oxide perovskites. As seen in Figure S11, the Pb–I atomic columns exhibit the highest contrast, while the contrast divergence between FA/Cs and I columns is relatively small due to the similar atomic numbers. With the angstrom-level real-space spatial resolution, the overlaps of atomic columns are well identified, which can be rarely achieved in the case of regular TEM imaging. For example, the overlapping of Pb and I atoms in the projection direction leads to the elongation of corresponding column spots to an oval shape. At the same time, the periodically variated tilt angle of the Pb–I column spots also accurately fits the structure model of orthorhombic CsPbI_3_, as shown in Figure 2a. The overlapping of two I atoms also makes the I column spots elongated. In addition, the measured average lattice spacing *c* of this orthorhombic FA-Cs MHP from the STEM-HAADF image and FFT pattern is 12.78 Å, larger than that of a 5 mol % FA-incorporated orthorhombic CsPbI_3_ sample, as shown in Figure 2d as well as that of pure CsPbI_3_ MHP in the literature (*a* = 8.646 Å, *b* = 8.818 Å, *c* = 12.52 Å).^44^

We further revealed an interesting atomic-scale lattice heterogeneity in this specimen, which is attributed to the nonuniform distribution of FA or Cs cations. Such heterogeneity is largely invisible to laboratory XRD measurements (Figure S1). In the left side of Figure 2b, the regular shape of adjacent FA/Cs and I columns in orthorhombic FA-Cs MHP is zigzag-like, and the corresponding signal intensity line profile demonstrates a higher contrast of FA/Cs columns with a higher atomic number (*Z*) in comparison to I columns (Figure 2c). However, a nanoscale cluster (about 2 × 2 nm size) was found with a variation in lattice shape in Figure 2a,b. From the enlarged atomic image of this cluster (Figure 2b), the adjacent FA/Cs and I columns tend to array along a straight line, as marked by the yellow arrow in the right side. Interestingly, a variation occurs in the signal intensity line profile (Figure 2c), which exhibits a slightly higher contrast of I columns in comparison to FA/Cs columns. This indicates a higher concentration of FA incorporation into the FA/Cs columns in this cluster, leading to a noticeable decrease in signal intensity in the *Z*-contrast image. This phenomenon is also consistent with the STEM-HAADF image simulation results on FA-Cs perovskites with different FA incorporation levels, as shown in Figure S12. Furthermore, an expansion of lattice spacing *c* was found in this FA-rich cluster (12.89 Å) in comparison with a regular Cs-rich region (12.80 Å), which was also consistent with a higher concentration of FA incorporation. Therefore, this nonuniform FA incorporation forms nanoscale FA-rich clusters with a tensile strain within MHP grains, creating numerous coherent, strained intragrain interfaces. Interestingly, with higher-level FA incorporation, not only does the average lattice spacing *c* keep increasing (Figure 2d–f) but also the in-plane strain (ε_*xx*_) distribution becomes more uneven with larger variations at the nanoscale (Figure 2g–i). This indicates a higher density of FA-rich clusters, as well as strained intragrain composition-boundary interfaces in FA-Cs perovskites with a FA cation content, that exceeds 50 mol %, when we examined a range of compositions (15–85 mol %). Interestingly, in comparison with FA-Cs MHP nanocrystals, which exhibit an almost complete FA-Cs segregation,^26^ the FA-Cs ion segregation is much slighter in 3D FA-Cs perovskites. For example, according to the STEM-HAADF simulation (Figure S12), the intensity variation of FA/Cs and I columns shown in Figure 2c may imply an increase in FA incorporation from about 15 mol % to 40 mol % across the composition–boundary interface.

**Figure 3:**
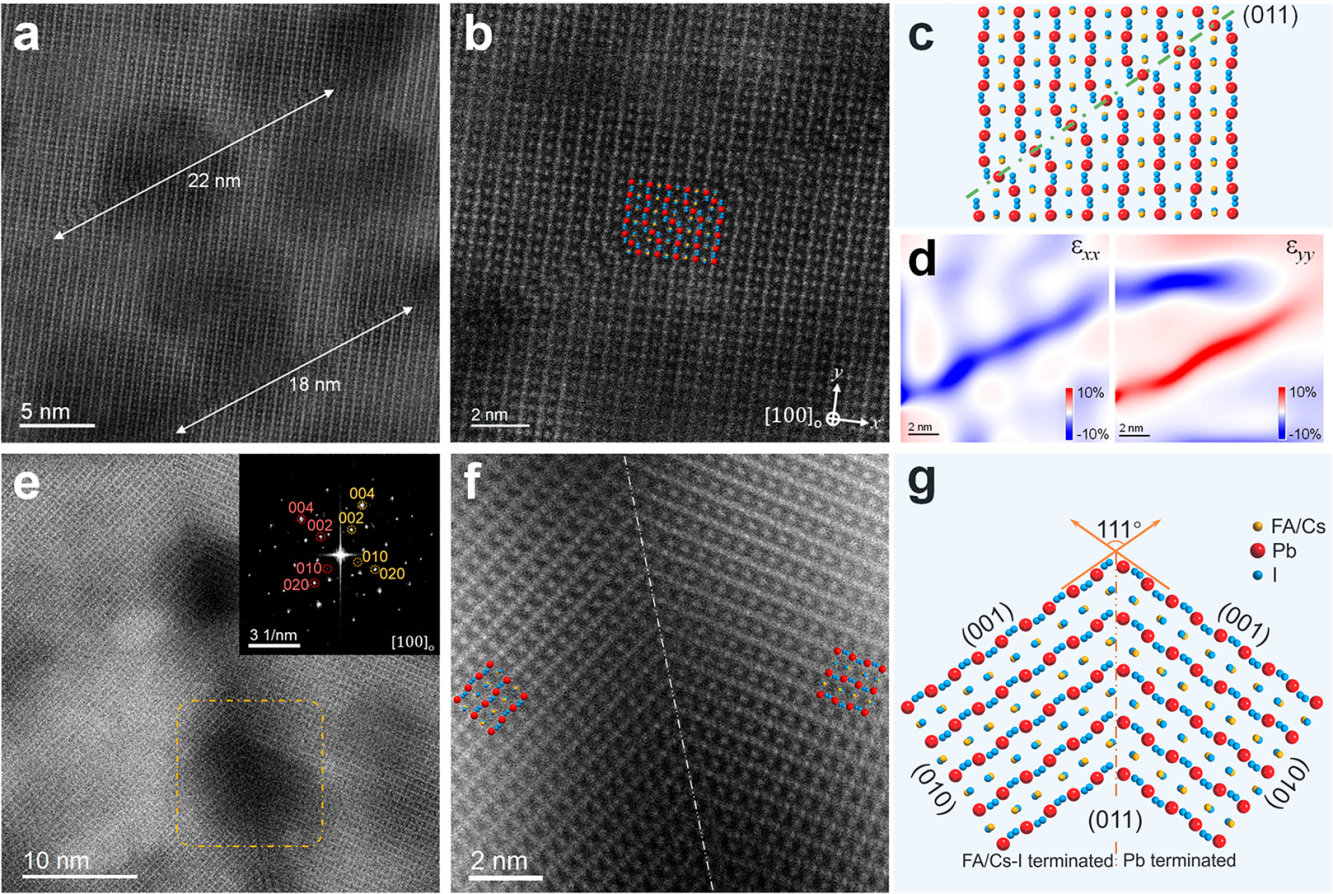
Atomic-scale structures of intragrain stacking-fault and twinning interfaces. (a) High-resolution unfiltered STEM-HAADF image of stacking faults in orthorhombic FA_0.5_Cs_0.5_PbI_3_ grains along the [100] projection direction. (b) Atomic-scale Butterworth filtered STEM-HAADF image of a single stacking fault, indicating the detailed atomic structure. (c) Reconstructed atomic model of the stacking fault in (b) along the (011) lattice plane. (d) In-plane strain ε_*xx*_ and ε_*yy*_ distribution of (b) generated by GPA analysis, showing a dramatic lattice distortion at the stacking fault region. (e) High-resolution unfiltered STEM-HAADF image of a typical twin boundary in FA_0.5_Cs_0.5_PbI_3_ grains. The upper right inset is the corresponding FFT pattern, showing a stacking of two twisted patterns along the [100] projection direction. (f) Atomic-scale Bragg filtered STEM-HAADF image from the yellow square region in (e) revealing the atomic details of this twin boundary. (g) Reconstructed atomic model of the twin boundary in (f) along the (011) lattice plane.

Another important finding is the observed fine structures of intragrain stacking faults in MHPs. In orthorhombic FA_0.5_Cs_0.5_PbI_3_ grains projected along the [100]_o_ direction (Figure S13), a typical kind of stacking fault formed by lattice plane displacement was identified (Figure 3a). From the atomic-scale STEM-HAADF image and reconstructed atomic model (Figure 3b,c), it is clear that this type of stacking fault follows the (011) lattice plane with a shared Pb layer. The stacking fault also breaks the local lattice periodicity and leads to a dramatic in-plane strain in both the *x* and *y* directions, as shown in Figure 3d. It is worth noting that these types of stacking faults have characteristic lengths of 8–25 nm and may occur with a higher prevalence in some regions rather than being uniformly distributed (Figure S14). These stacking faults are similar to those reported by Rothmann et al. in pure FAPbI_3_,^13^ but the fault is along a series of lead columns rather than iodide columns, and the shift across the fault is more than three-fourths of a unit cell rather than half a unit cell in pure FAPbI_3_. This illustrates how slight changes in chemistry can lead to significant changes in crystallography, further highlighting the importance of a careful characterization of new perovskite materials.

We then examined intragrain twin-boundary interfaces in FA-Cs MHPs. The targeted twin boundary to observe is typical and is aligned throughout the film thickness (Figures S15 and S17). The FFT pattern of the high-resolution STEM-HAADF image taken from the twin-boundary region consists of two arrays of twisted independent patterns, which also correspond to an orthorhombic lattice structure but along [100]_o_ (Figure 3e and Figure S13). The improvements in imaging enabled by our approach are critical, as the detailed atomic structures of this twin boundary and adjacent MHP lattices are now revealed (Figure 3f and Figures S16–S17). Thus, the lattice structures on both sides of the twin boundary are revealed to be highly symmetrical and consistent with the atomic model of a [100]_o_-projected orthorhombic MHP, in good agreement with the FFT results. This enables us to precisely reconstruct the real atomic structure. As illustrated in Figure 3g, lattices on both sides are terminated at this twin boundary which follows the (011) lattice plane. In addition, the termination layers of these adjacent regions are not the same. The left terminates at the FA/Cs–I layer, while the right terminates at the Pb layer. This difference leads to the breaking of symmetry at these twin boundaries in FA-Cs PSCs. To further validate the atomic structure of these intragrain twin boundaries, continuous electron probe scanning was used to trigger damage in the twin-boundary region, as shown in Figure S18. We found that, even as e-beam damage occurs at the core region, the MHP structure is retained without a phase transformation or decomposition, and the twin-boundary structure remains unchanged as well (Figure S18b). On the basis of these detailed structural studies, we are then able to harness these results to undertake calculations to understand the implications on the electronic properties, which are striking.

### DFT Calculations and Electronic Structures of Intragrain Interfaces

**Figure 4:**
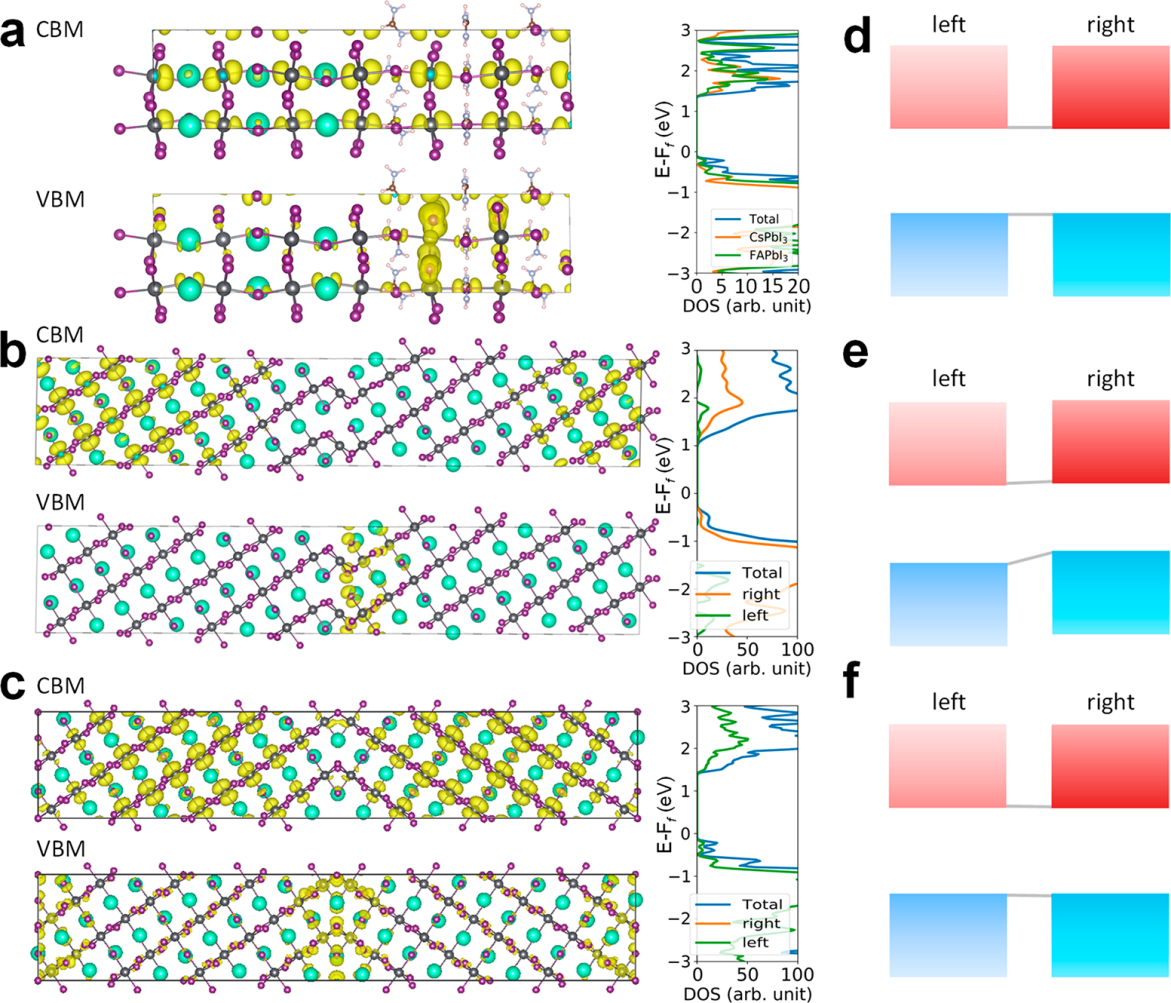
Electronic structures of the three prototypical intragrain interfaces. The charge density corresponding to the VBM and CBM, the total DOS, and partial DOS of the left and right domains of the (a) composition-boundary interface, (b) stacking-fault interface, and (c) twin-boundary interface. The cyan, black, and purple spheres denote Cs, Pb, and I atoms, respectively, and the yellow areas denote the computed charge densities. (d–f) Band alignment diagrams for the three intragrain interfaces, where the upper red rectangles represent CBM while the lower blue rectangles represent VBM. Note that for (d), the left and right electronic structures are based on relaxed CsPbI_3_ (left grain) and FAPbI_3_ (right grain) shown in (a), respectively.

With the detailed atomic information acquired via the STEM-HAADF imaging, we constructed equivalent atomistic models for the three prototypical intragrain interfaces, as shown in Figures 4a–c. For simplification, pure CsPbI_3_ and FAPbI_3_ models were employed to represent the Cs-rich and FA-rich regions that are observed under STEM, respectively. The composition-boundary interface is simulated using a superlattice model formed by stacking six CsI/PbI_2_ (left) and six FAI/PbI_2_ (right) atomic layers, respectively, while these layers are perpendicular to the [001] axis (see Figure 4a). The stacking-fault interface was built using the builder module implemented in the pymatgen package,^45^ where [100] and (011) were used as the rotation axis and interface plane, respectively, while the rotation angle was set to 0° and an in-plane shift along the (011) plane by 0.2 unit (Figure 4b) was used, followed by relocating one Pb atom slightly in the interface to match the apparent central Pb atom in the interface, as shown in Figure 3b,c. After geometric optimization (Figure 4b), an interesting reconstruction of the local Pb–I bonding pattern occurs in the interfacial layer (see Figure S19 for more details). Such a reconstruction of the local Pb–I bonding pattern may also occur in the realistic atomic structure of stacking faults, as shown in Figure 3b,c. The atomic structure of the twin-boundary interface was constructed by using a mirror operator along the (011) plane (Figure 4c); as a result, the Pb–I octahedra at the interface were connected through shared common faces.

The computed electronic structure and density of states (DOS) of the composition-boundary interface are shown in Figure 4a. The valence band maximum (VBM) is mainly composed of the antibonding I p states and Pb s states, while the conduction band minimum (CBM) is a hybrid of the p states of I and Pb with a majority having a nonbonding character and a minority having an antibonding nature,^46^ as shown from the computed charge density (Figure 4a); these states are from both near the interface and inside the grains. On the basis of the partial DOS of the CsPbI_3_ part (orange curve) and FAPbI_3_ part (green curve) (Figure 4a), we found that the band edges of both contributions do not exhibit a mismatch with each other, as illustrated in Figure 4d. In addition, we found a decrease in the band gap for the composition-boundary interface, in comparison to that for pristine CsPbI_3_ (1.7 eV from the PBE DFT computation). This is due to tilting of local octahedra, which enhances antibonding interactions between Pb s states and I p states (see the denser yellow areas near the interface in the lower panel of Figure 4a). Overall, the computed DOS does not show any gap states, indicating the benign electronic properties of the composition-boundary interface on the electronic gap.

For the stacking-fault interface, we found that the bonding p states of the I and *s* state of Pb sites near the interface shift to higher energy in comparison to the states inside the grain, while the CBM is dominated by the Pb p states contributed by both the interior domain and stacking-fault interface. The partial DOS (Figure 4b) of the left and right domains indicate a quasi-type-II band alignment (Figure 4e). This quasi-type-II aligned electronic structure may facilitate the dissociation of the photogenerated excitons and separation of charge carriers, imparting beneficial effects to the PSC performance.

With regard to the twin-boundary interface, we found that the rotation angle of the optimized interface model is 110.7°, in very good agreement with the experimental observation, as shown in Figure 3g. Similar to the other types of interfaces, the DOS of the twin-boundary interface exhibit negligible gap states, as shown in Figure 4c. The VBM is again mainly from the antibonding I p states and partially from the Pb s states near the interface, while the CBM is dominated by the Pb p states inside the grains (see yellow areas in Figure 4c). The partial DOS of the left and right domains are almost identical with each other (see the schematic band alignment in Figure 4f), since the two domains are the mirror image of each other. As a result, we can conclude that, in general, the twin-boundary interface incurs little detrimental effects on the PSC performance from the perspective of electronic structure. However, it is worth noting that, in both composition-boundary and twin-boundary interfaces, the inversion symmetry is broken. Therefore, we performed additional computation of the electronic density of states for these two interface models, for which the PBE+SOC (spin–orbit coupling) calculation was undertaken. The computation results shown in Figures S20 and S21 suggest that the electronic band gap is reduced (in comparison to the PBE calculation result shown in Figure 4a,c).

## Discussion

**Figure 5:**
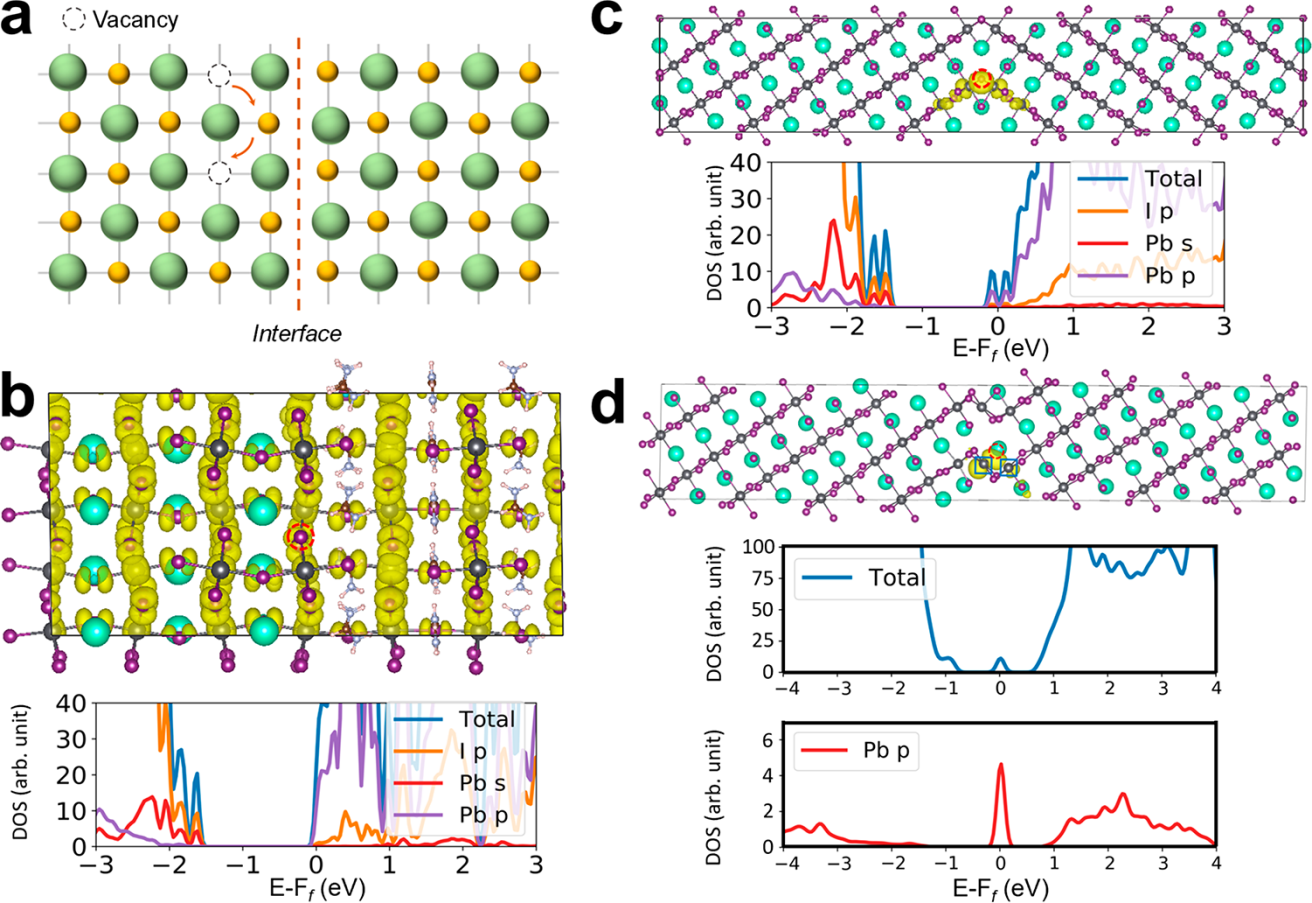
Electronic structures of the three prototypical intragrain interfaces. (a) Schematic illustration of the interaction of I^–^ vacancies with three types of interfaces. (b, c) Charge density (yellow area) of the shoulder peak near the band edges, total DOS, and partial DOS for Pb s and p orbitals and I p orbitals for (b) the composition-boundary interface with one I vacancy (per supercell) and (c) the twin-boundary interface with one I vacancy. (d) Charge density (yellow area) of the trap state below the Fermi level (upper panel), total DOS (middle panel), and partial DOS (lower panel) of selected Pb p orbitals at the stacking-fault interface. The selected Pb atoms are highlighted by two blue squares. In (b–d) the I^–^ vacancy per supercell is highlighted by the red dashed circle.

We note that these intragrain interfaces in MHPs are not entirely static upon the external stimuli. As schematically shown in Figure 5a, they can serve as “sinks” for point defects. In MHPs, point defects such as I vacancies are known to be highly mobile due to low activation energy barriers.^47,48^ Such potential interactions can induce new influences on the electronic structure and properties of MHPs.^49−52^ To illustrate the effect of a point defect-interface interaction on the electronic structures of the interfaces, we considered the addition of I vacancies into the composition-boundary and twin-boundary interfaces, respectively, as shown in Figure 5b,c. Notably, the resultant DOS plots immediately show sharp peaks near the valence band edges. Nevertheless, from the charge density plots (as marked by yellow areas), we observed that the peak states are mainly due to the p states of I atoms inside the grains, which are still considered benign.

On the other hand, because of the grain shift and Pb atom relocation at the stacking-fault interface, the local Pb–I bonding pattern stemming from the relocated Pb atoms (e.g., Pb0 and Pb0′ in Figure S19) and its neighboring I atoms is changed within the interface, where the nearest neighboring Pb–Pb distance is notably shortened and the number of Pb–I bonds is increased from two to five in comparison to those in the bulk crystal (Figure S19). After an I vacancy (at the location of I1) is created, the computed total and partial DOS (Figure 5d, middle and lower panels) confirm that the localized states (deep trap states) below the conduction band edge are due to the I vacancy (I1 in Figure S19). Note that I1 forms the shortest Pb–I bond (∼3.0 Å) with the relocated Pb atom (Pb0). The localization of these states can be also visualized from the charge density, where the p characteristics due to the I vacancy can be discerned.

We also introduced Pb interstitials into the twin-boundary interface, as shown in Figure S22. It is found that the excess Pb indirectly leads to localized trap states that are mainly contributed by the p orbitals of Pb and I atoms close to the defect, as evidenced by the charge density and the projected DOS (see Figure S22). Clearly, these trap states will be detrimental to the device’s performance and should be avoided as much as possible to maximize the PES. Indeed, the strategy and physics of reducing the point defects are in line with those for developing a low-dimensional MHP and surface defect passivation.

Atomic-scale structural information on intragrain interfaces is unambiguously attained on the basis of a reliable STEM characterization approach in high-performance MHPs, facilitating the construction of accurate interface models for the theoretical investigation of emergent electronic properties. The combination of detailed physical structural data to inform a theory-based electronic structure permits us to show how these prototypical intragrain interfaces exhibit relatively benign electronic properties regardless of their population. This understanding also informs critical technological questions relating to the interaction of mobile I-vacancy defects with the stacking-fault interface and the mobile Pb-interstitial defects that can evoke detrimental effects if they are not controlled. This work then begins to provide a mechanistic foundation for many of the interface engineering approaches within these systems that drive further device improvement.

## Experimental Section

### MHP Precursor Solution and Film Synthesis

The FA-Cs mixed MHP (FA_*x*_Cs_1–*x*_PbI_3_) thin films have been prepared according to the method reported previously.^37^ First, 1.3 M MHP precursor solutions of FA_*x*_Cs_1–*x*_PbI_3_ were prepared by dissolving FAI, CsI, and PbI_2_ with ratios of 1:*x*:1 in dimethyl sulfoxide (DMSO). The precursor solutions were stirred at 65 °C for 6 h to be ready for use. Then, the precursor solutions were spin-coated on substrates using a programmed two-step process: 1000 rpm for 10 s as the first step and 3000 rpm for 30 s as the second step. 360 μL of ethyl acetate (antisolvent) was dropped in the center of the spinning films at 10 s after the start of the second step. The as-spun thin films were heated at 100 °C for 5 min and then at 220 °C for 12 min to form FA_*x*_Cs_1–*x*_PbI_3_ MHP thin films.

### PSC Device Fabrication

To fabricate PSCs, a patterned FTO glass substrate was cleaned by ultrasonic washing successively in saturated KOH isopropanol solution, deionized water, and ethanol. Then, a 10 nm thick compact TiO_2_ hole-blocking layer was deposited by atomic layer deposition (ALD). Subsequently, the MHP thin films were deposited according to the method mentioned above. Then, an HTM layer was deposited by spin-coating a stock solution of Spiro-OMeTAD at 3000 rpm for 30 s. The Spiro-OMeTAD solution was prepared by dissolving 72.3 mg of Spiro-OMeTAD in 1 mL of chlorobenzene, to which 28.8 μL of 4-*tert*-butylpyridine and 17.5 μL of lithium bis(trifluoromethanesulfonyl) imide (LITSFI) solution (520 mg of LITSFI in 1 mL of acetonitrile) was added. Finally, a 80 nm thick Au top electrode was deposited via thermal evaporation to complete the PSC device.

### STEM Specimen Preparation

The cross-sectional TEM specimens of PSCs were prepared by a dual-beam focused ion beam (FIB) nanofabrication platform (Helios 600i, Thermofisher, USA). A protecting layer was first deposited on the top surface of the devices by electron deposition of Pt, followed by etching the surrounding area to form the specimen lamella. The operating voltage of the gallium ion beam was 30 kV, and the working current was 0.1–24 nA for lamella processing. The lamella was then lifted out from the substrate and transferred *in situ* to a TEM half-grid inside the FIB chamber. The observation area of lamella was thinned to less than 100 nm with a 40–790 pA gallium ion beam. To minimize the damage induced by ion implantation to sample lamella, a fine milling and polishing process was adopted by using a gallium ion beam with an accelerating voltage down to 1 kV and 72 pA working current to remove the surface amorphous layer. After the FIB preparation and polishing procedures, the as-prepared cross-sectional PSC specimens were transferred to a high-vacuum sputter coater for protecting layer deposition. Amorphous carbon layers with a thickness of 10 nm were coated on both sides of the cross-section specimen using pulsed carbon evaporation at 8 × 10^–5^ mbar.

### STEM Characterization

STEM observations of the device cross-section specimens were carried out with a regular aberration-corrected STEM microscope (Titan G2 60-300, Thermofisher, USA; Equipped with a field emission gun) with a 300 kV electron beam accelerating voltage. The beam current of the electron probe was reduced to 5 pA to minimize the damage to MHP frameworks during atomic resolution imaging. The probe convergence angle was 24.5 mrad, and the angular range of the HAADF detector was from 79.5 to 200 mrad. The dwell time of each pixel during STEM-HAADF image acquisition was 6 μs, and the size of all STEM-HAADF images in this work was 2048 × 2048 pixel^2^. For the typical high-resolution STEM-HAADF images illustrated in this work, the frame size was 34.5 × 34.5 nm^2^. The total electron dose was 1.3 × 10^4^ e Å^2^ for the acquisition of a single STEM-HAADF image.

### DFT Calculations

Density functional theory (DFT) calculations were performed using the generalized gradient approximation (GGA) in the Perdew–Burke–Ernzerhof (PBE) format, as implemented in the Vienna *ab initio* simulation package (VASP 5.4). The projector augmented wave method (PAW) was used to describe the interaction between core electrons and valence electrons. In particular, a kinetic energy cutoff for the plane-wave basis was set to 450 eV. Specifically, 5s^2^5p^6^6s of Cs, 6s^2^6p^2^ of Pb, and 5s^2^5p^5^ of I were used as valence electrons. Grimme’s DFT-D3 correction was adopted to describe the long-range van der Waals interactions. The stacking-fault interface was created using the builder module in the pymatgen package,^45^ where each unit grain was expanded by a factor of 2 to exclude the interaction between adjacent interfaces. A γ point was adopted for the structure optimization, and both atomic positions and lattice constants were relaxed until the residual forces on atoms were less than 0.02 eV/Å and the total energy change was less than 5 × 10^–5^ eV while the lattice angles were fixed. Denser 2 × 2 × 1, 2 × 1 × 1, and 2 × 1 × 1 *k*-point meshes were used in the electronic property calculations for the composition, twin, and stacking fault grain interfaces, respectively. Furthermore, the electronic properties of composition-boundary and twin-boundary interfaces were calculated with consideration of the spin–orbit coupling (SOC) effect.

## Supporting Information Available

STEM image filtering, geometric phase analysis (GPA) strain mapping, device characterization and testing, and other additional figures as described in the text (PDF)

## References

[ref1] Dunlap-Shohl W. A.; Zhou Y.; Padture N. P.; Mitzi D. B. Synthetic Approaches for Halide Perovskite Thin Films. Chem. Rev. 2019, 119, 3193–3295. 10.1021/acs.chemrev.8b00318. 30387358

[ref2] Correa-Baena J.-P.; Saliba M.; Buonassisi T.; Grätzel M.; Abate A.; Tress W.; Hagfeldt A. Promises and challenges of perovskite solar cells. Science 2017, 358, 739–744. 10.1126/science.aam6323. 29123060

[ref3] Best Research-Cell Efficiency Chart: nrel.gov/pv/cell-efficiency.html, accessed Dec 19, 2021.

[ref4] Jung E. H.; Jeon N. J.; Park E. Y.; Moon C. S.; Shin T. J.; Yang T. Y.; Noh J. H.; Seo J. Efficient, stable and scalable perovskite solar cells using poly(3-hexylthiophene). Nature 2019, 567, 511–515. 10.1038/s41586-019-1036-3. 30918371

[ref5] Jiang Q.; Zhao Y.; Zhang X.; Yang X.; Chen Y.; Chu Z.; Ye Q.; Li X.; Yin Z.; You J. Surface passivation of perovskite film for efficient solar cells. Nat. Photonics 2019, 13, 460–466. 10.1038/s41566-019-0398-2.

[ref6] Jeong J.; Kim M.; Seo J.; Lu H.; Ahlawat P.; Mishra A.; Yang Y.; Hope M. A.; Eickemeyer F. T.; Kim M.; Yoon Y. J.; Choi I. W.; Darwich B. P.; Choi S. J.; Jo Y.; Lee J. H.; Walker B.; Zakeeruddin S. M.; Emsley L.; Rothlisberger U.; Hagfeldt A.; Kim D. S.; Gratzel M.; Kim J. Y. Pseudo-halide anion engineering for alpha-FAPbI_3_ perovskite solar cells. Nature 2021, 592, 381–385. 10.1038/s41586-021-03406-5. 33820983

[ref7] Liu Z.; Qiu L.; Ono L. K.; He S.; Hu Z.; Jiang M.; Tong G.; Wu Z.; Jiang Y.; Son D.-Y.; Dang Y.; Kazaoui S.; Qi Y. A holistic approach to interface stabilization for efficient perovskite solar modules with over 2,000-h operational stability. Nature Energy 2020, 5, 596–604. 10.1038/s41560-020-0653-2.

[ref8] Zheng X.; Hou Y.; Bao C.; Yin J.; Yuan F.; Huang Z.; Song K.; Liu J.; Troughton J.; Gasparini N.; Zhou C.; Lin Y.; Xue D.-J.; Chen B.; Johnston A. K.; Wei N.; Hedhili M. N.; Wei M.; Alsalloum A. Y.; Maity P.; Turedi B.; Yang C.; Baran D.; Anthopoulos T. D.; Han Y.; Lu Z.-H.; Mohammed O. F.; Gao F.; Sargent E. H.; Bakr O. M. Managing grains and interfaces via ligand anchoring enables 22.3%-efficiency inverted perovskite solar cells. Nature Energy 2020, 5, 131–140. 10.1038/s41560-019-0538-4.

[ref9] Christians J. A.; Schulz P.; Tinkham J. S.; Schloemer T. H.; Harvey S. P.; Tremolet de Villers B. J.; Sellinger A.; Berry J. J.; Luther J. M. Tailored interfaces of unencapsulated perovskite solar cells for > 1,000 h operational stability. Nature Energy 2018, 3, 68–74. 10.1038/s41560-017-0067-y.

[ref10] Saidaminov M. I.; Williams K.; Wei M.; Johnston A.; Quintero-Bermudez R.; Vafaie M.; Pina J. M.; Proppe A. H.; Hou Y.; Walters G.; Kelley S. O.; Tisdale W. A.; Sargent E. H. Multi-cation perovskites prevent carrier reflection from grain surfaces. Nat. Mater. 2020, 19, 412–418. 10.1038/s41563-019-0602-2. 32042078

[ref11] de Quilettes D. W.; Vorpahl S. M.; Stranks S. D.; Nagaoka H.; Eperon G. E.; Ziffer M. E.; Snaith H. J.; Ginger D. S. Impact of microstructure on local carrier lifetime in perovskite solar cells. Science 2015, 348, 683–686. 10.1126/science.aaa5333. 25931446

[ref12] Park J.-S.; Walsh A. Modeling Grain Boundaries in Polycrystalline Halide Perovskite Solar Cells. Annual Review of Condensed Matter Physics 2021, 12, 95–109. 10.1146/annurev-conmatphys-042020-025347.

[ref13] Rothmann M. U.; Kim J. S.; Borchert J.; Lohmann K. B.; O’Leary C. M.; Sheader A. A.; Clark L.; Snaith H. J.; Johnston M. B.; Nellist P. D.; Herz L. M. Atomic-scale microstructure of metal halide perovskite. Science 2020, 370, eabb5940 10.1126/science.abb5940. 33122356

[ref14] Rothmann M. U.; Li W.; Etheridge J.; Cheng Y. B. Microstructural Characterisations of Perovskite Solar Cells–From Grains to Interfaces: Techniques, Features, and Challenges. Adv. Energy Mater. 2017, 7, 1700912 10.1002/aenm.201700912.

[ref15] Xiao X.; Li W.; Fang Y.; Liu Y.; Shao Y.; Yang S.; Zhao J.; Dai X.; Zia R.; Huang J. Benign ferroelastic twin boundaries in halide perovskites for charge carrier transport and recombination. Nat. Commun. 2020, 11, 2215 10.1038/s41467-020-16075-1. 32371861 PMC7200693

[ref16] Song J.; Zhou Y.; Padture N. P.; Huey B. D. Anomalous 3D nanoscale photoconduction in hybrid perovskite semiconductors revealed by tomographic atomic force microscopy. Nat. Commun. 2020, 11, 3308 10.1038/s41467-020-17012-y. 32620841 PMC7335063

[ref17] MacDonald G. A.; Yang M.; Berweger S.; Killgore J. P.; Kabos P.; Berry J. J.; Zhu K.; DelRio F. W. Methylammonium lead iodide grain boundaries exhibit depth-dependent electrical properties. Energy Environ. Sci. 2016, 9, 3642–3649. 10.1039/C6EE01889K.

[ref18] Stavrakas C.; Zhumekenov A. A.; Brenes R.; Abdi-Jalebi M.; Bulovic V.; Bakr O. M.; Barnard E. S.; Stranks S. D. Probing buried recombination pathways in perovskite structures using 3D photoluminescence tomography. Energy Environ. Sci. 2018, 11, 2846–2852. 10.1039/C8EE00928G. 30713582 PMC6333269

[ref19] Li W.; Rothmann M. U.; Zhu Y.; Chen W.; Yang C.; Yuan Y.; Choo Y. Y.; Wen X.; Cheng Y.-B.; Bach U.; Etheridge J. The critical role of composition-dependent intragrain planar defects in the performance of MA_1–x_FA_x_PbI_3_ perovskite solar cells. Nature Energy 2021, 6, 624–632. 10.1038/s41560-021-00830-9.

[ref20] Yin W. J.; Shi T.; Yan Y. Unique properties of halide perovskites as possible origins of the superior solar cell performance. Adv. Mater. 2014, 26, 4653–4658. 10.1002/adma.201306281. 24827122

[ref21] Ji D.; Cai S.; Paudel T. R.; Sun H.; Zhang C.; Han L.; Wei Y.; Zang Y.; Gu M.; Zhang Y.; Gao W.; Huyan H.; Guo W.; Wu D.; Gu Z.; Tsymbal E. Y.; Wang P.; Nie Y.; Pan X. Freestanding crystalline oxide perovskites down to the monolayer limit. Nature 2019, 570, 87–90. 10.1038/s41586-019-1255-7. 31168106

[ref22] Cai S.; Zhou Y. Visualizing the Invisible in Perovskites. Joule 2020, 4, 2545–2548. 10.1016/j.joule.2020.11.014.

[ref23] Aguiar J. A.; Alkurd N. R.; Wozny S.; Patel M. K.; Yang M.; Zhou W.; Al-Jassim M.; Holesinger T. G.; Zhu K.; Berry J. J. In situ investigation of halide incorporation into perovskite solar cells. MRS Commun. 2017, 7, 575–582. 10.1557/mrc.2017.52.

[ref24] Aguiar J. A.; Wozny S.; Alkurd N. R.; Yang M.; Kovarik L.; Holesinger T. G.; Al-Jassim M.; Zhu K.; Zhou W.; Berry J. J. Effect of Water Vapor, Temperature, and Rapid Annealing on Formamidinium Lead Triiodide Perovskite Crystallization. ACS Energy Letters 2016, 1, 155–161. 10.1021/acsenergylett.6b00042.

[ref25] Thind A. S.; Luo G.; Hachtel J. A.; Morrell M. V.; Cho S. B.; Borisevich A. Y.; Idrobo J. C.; Xing Y.; Mishra R. Atomic Structure and Electrical Activity of Grain Boundaries and Ruddlesden-Popper Faults in Cesium Lead Bromide Perovskite. Adv. Mater. 2019, 31, 1805047 10.1002/adma.201805047. 30506822

[ref26] Hao M.; Bai Y.; Zeiske S.; Ren L.; Liu J.; Yuan Y.; Zarrabi N.; Cheng N.; Ghasemi M.; Chen P.; Lyu M.; He D.; Yun J.-H.; Du Y.; Wang Y.; Ding S.; Armin A.; Meredith P.; Liu G.; Cheng H.-M.; Wang L. Ligand-assisted cation-exchange engineering for high-efficiency colloidal Cs_1–x_FA_x_PbI_3_ quantum dot solar cells with reduced phase segregation. Nature Energy 2020, 5, 79–88. 10.1038/s41560-019-0535-7.

[ref27] Zhu Y.; Gui Z.; Wang Q.; Meng F.; Feng S.; Han B.; Wang P.; Huang L.; Wang H.-L.; Gu M. Direct atomic scale characterization of the surface structure and planar defects in the organic-inorganic hybrid CH_3_NH_3_PbI_3_ by Cryo-TEM. Nano Energy 2020, 73, 104820 10.1016/j.nanoen.2020.104820.

[ref28] Li Y.; Zhou W.; Li Y.; Huang W.; Zhang Z.; Chen G.; Wang H.; Wu G.-H.; Rolston N.; Vila R.; Chiu W.; Cui Y. Unravelling Degradation Mechanisms and Atomic Structure of Organic-Inorganic Halide Perovskites by Cryo-EM. Joule 2019, 3, 2854–2866. 10.1016/j.joule.2019.08.016. 34109301 PMC8186345

[ref29] Zhou Y.; Sternlicht H.; Padture N. P. Transmission Electron Microscopy of Halide Perovskite Materials and Devices. Joule 2019, 3, 641–661. 10.1016/j.joule.2018.12.011.

[ref30] Divitini G.; Cacovich S.; Matteocci F.; Cinà L.; Di Carlo A.; Ducati C. In situ observation of heat-induced degradation of perovskite solar cells. Nature Energy 2016, 1, 15012 10.1038/nenergy.2015.12.

[ref31] Lu H.; Liu Y.; Ahlawat P.; Mishra A.; Tress W. R.; Eickemeyer F. T.; Yang Y.; Fu F.; Wang Z.; Avalos C. E.; Carlsen B. I.; Agarwalla A.; Zhang X.; Li X.; Zhan Y.; Zakeeruddin S. M.; Emsley L.; Rothlisberger U.; Zheng L.; Hagfeldt A.; Grätzel M. Vapor-assisted deposition of highly efficient, stable black-phase FAPbI_3_ perovskite solar cells. Science 2020, 370, eabb8985 10.1126/science.abb8985. 33004488

[ref32] Jeong M.; Choi I. W.; Go E. M.; Cho Y.; Kim M.; Lee B.; Jeong S.; Jo Y.; Choi H. W.; Lee J.; Bae J.-H.; Kwak S. K.; Kim D. S.; Yang C. Stable perovskite solar cells with efficiency exceeding 24.8% and 0.3-V voltage loss. Science 2020, 369, 1615 10.1126/science.abb7167. 32973026

[ref33] Deng Y.; Xu S.; Chen S.; Xiao X.; Zhao J.; Huang J. Defect compensation in formamidinium–caesium perovskites for highly efficient solar mini-modules with improved photostability. Nature Energy 2021, 6, 633–641. 10.1038/s41560-021-00831-8.

[ref34] Yang Z.; Zhang W.; Wu S.; Zhu H.; Liu Z.; Liu Z.; Jiang Z.; Chen R.; Zhou J.; Lu Q.; Xiao Z.; Shi L.; Chen H.; Ono L. K.; Zhang S.; Zhang Y.; Qi Y.; Han L.; Chen W. Slot-die coating large-area formamidinium-cesium perovskite film for efficient and stable parallel solar module. Science Advances 2021, 7, eabg3749 10.1126/sciadv.abg3749. 33931458 PMC8087413

[ref35] Turren-Cruz S.-H.; Hagfeldt A.; Saliba M. Methylammonium-free, high-performance, and stable perovskite solar cells on a planar architecture. Science 2018, 362, 449 10.1126/science.aat3583. 30309904

[ref36] Masi S.; Gualdrón-Reyes A. F.; Mora-Seró I. Stabilization of Black Perovskite Phase in FAPbI_3_ and CsPbI_3_. ACS Energy Letters 2020, 5, 1974–1985. 10.1021/acsenergylett.0c00801.

[ref37] Shao Z.; Meng H.; Du X.; Sun X.; Lv P.; Gao C.; Rao Y.; Chen C.; Li Z.; Wang X.; Cui G.; Pang S. Cs_4_PbI_6_-Mediated Synthesis of Thermodynamically Stable FA_0.15_Cs_0.85_PbI_3_ Perovskite Solar Cells. Adv. Mater. 2020, 32, 2001054 10.1002/adma.202001054. 32567102

[ref38] Krivanek O. L.; Chisholm M. F.; Nicolosi V.; Pennycook T. J.; Corbin G. J.; Dellby N.; Murfitt M. F.; Own C. S.; Szilagyi Z. S.; Oxley M. P.; Pantelides S. T.; Pennycook S. J. Atom-by-atom structural and chemical analysis by annular dark-field electron microscopy. Nature 2010, 464, 571–574. 10.1038/nature08879. 20336141

[ref39] Tang Y. L.; Zhu Y. L.; Ma X. L.; Borisevich A. Y.; Morozovska A. N.; Eliseev E. A.; Wang W. Y.; Wang Y. J.; Xu Y. B.; Zhang Z. D.; Pennycook S. J. Observation of a periodic array of flux-closure quadrants in strained ferroelectric PbTiO_3_ films. Science 2015, 348, 547 10.1126/science.1259869. 25883317

[ref40] Song K.; Liu L.; Zhang D.; Hautzinger M. P.; Jin S.; Han Y. Atomic-Resolution Imaging of Halide Perovskites Using Electron Microscopy. Adv. Energy Mater. 2020, 10, 1904006 10.1002/aenm.201904006.

[ref41] Alberti A.; Bongiorno C.; Smecca E.; Deretzis I.; La Magna A.; Spinella C. Pb clustering and PbI_2_ nanofragmentation during methylammonium lead iodide perovskite degradation. Nat. Commun. 2019, 10, 2196 10.1038/s41467-019-09909-0. 31097719 PMC6522562

[ref42] Chen S.; Zhang Y.; Zhao J.; Mi Z.; Zhang J.; Cao J.; Feng J.; Zhang G.; Qi J.; Li J.; Gao P. Transmission electron microscopy of organic-inorganic hybrid perovskites: myths and truths. Science Bulletin 2020, 65, 1643–1649. 10.1016/j.scib.2020.05.020. 36659040

[ref43] Chen S.; Zhang Y.; Zhang X.; Zhao J.; Zhao Z.; Su X.; Hua Z.; Zhang J.; Cao J.; Feng J.; Wang X.; Li X.; Qi J.; Li J.; Gao P. General Decomposition Pathway of Organic-Inorganic Hybrid Perovskites through an Intermediate Superstructure and its Suppression Mechanism. Adv. Mater. 2020, 32, 2001107 10.1002/adma.202001107. 32419179

[ref44] Zhao B.; Jin S. F.; Huang S.; Liu N.; Ma J. Y.; Xue D. J.; Han Q.; Ding J.; Ge Q. Q.; Feng Y.; Hu J. S. Thermodynamically Stable Orthorhombic gamma-CsPbI_3_ Thin Films for High-Performance Photovoltaics. J. Am. Chem. Soc. 2018, 140, 11716–11725. 10.1021/jacs.8b06050. 30153411

[ref45] Ong S. P.; Richards W. D.; Jain A.; Hautier G.; Kocher M.; Cholia S.; Gunter D.; Chevrier V. L.; Persson K. A.; Ceder G. Python Materials Genomics (pymatgen): A robust, open-source python library for materials analysis. Comput. Mater. Sci. 2013, 68, 314–319. 10.1016/j.commatsci.2012.10.028.

[ref46] Prasanna R.; Gold-Parker A.; Leijtens T.; Conings B.; Babayigit A.; Boyen H. G.; Toney M. F.; McGehee M. D. Band Gap Tuning via Lattice Contraction and Octahedral Tilting in Perovskite Materials for Photovoltaics. J. Am. Chem. Soc. 2017, 139, 11117–11124. 10.1021/jacs.7b04981. 28704048

[ref47] Hussain F.; Hayat S. S.; Shah Z. A.; Hassan N.; Ahmad S. A. Interaction of point defects with twin boundaries in Au: A molecular dynamics study. Chinese Physics B 2013, 22, 096102 10.1088/1674-1056/22/9/096102.

[ref48] Suzuki A.; Mishin Y. Interaction of Point Defects with Grain Boundaries in fcc Metals. Interface Science 2003, 11, 425–437. 10.1023/A:1026195911339.

[ref49] deQuilettes D. W.; Koch S.; Burke S.; Paranji R. K.; Shropshire A. J.; Ziffer M. E.; Ginger D. S. Photoluminescence Lifetimes Exceeding 8 μs and Quantum Yields Exceeding 30% in Hybrid Perovskite Thin Films by Ligand Passivation. ACS Energy Letters 2016, 1, 438–444. 10.1021/acsenergylett.6b00236.

[ref50] Nenon D. P.; Pressler K.; Kang J.; Koscher B. A.; Olshansky J. H.; Osowiecki W. T.; Koc M. A.; Wang L. W.; Alivisatos A. P. Design Principles for Trap-Free CsPbX_3_ Nanocrystals: Enumerating and Eliminating Surface Halide Vacancies with Softer Lewis Bases. J. Am. Chem. Soc. 2018, 140, 17760–17772. 10.1021/jacs.8b11035. 30501174

[ref51] Moia D.; Maier J. Ion Transport, Defect Chemistry, and the Device Physics of Hybrid Perovskite Solar Cells. ACS Energy Letters 2021, 6, 1566–1576. 10.1021/acsenergylett.1c00227.

[ref52] Yin W.-J.; Shi T.; Yan Y. Unusual defect physics in CH_3_NH_3_PbI_3_ perovskite solar cell absorber. Appl. Phys. Lett. 2014, 104, 063903 10.1063/1.4864778.

