# Supplementary material for: Atomically Resolved Electrically Active Intragrain Interfaces in Perovskite Semiconductors
Source: J Am Chem Soc. 2022 Jan 21;144(4):1910–20. doi: 10.1021/jacs.1c12235 (PMC8815067; doi:10.1021/jacs.1c12235)
Supplement: Supplementary file 1 — ja1c12235_si_001.pdf [file ja1c12235_si_001.pdf]

Supplementary Information

Atomically Resolved Electrically Active Intragrain Interfaces in Perovskite Semiconductors

Songhua Cai[1 †, *], Jun Dai[2, †], Zhipeng Shao[3], Mathias Uller Rothmann[4], Yinglu Jia[2], Caiyun Gao[3], Mingwei Hao[5], Shuping Pang[3], Peng Wang [6, 7], Shu Ping Lau[1], Kai Zhu[8], Joseph J. Berry[9,10], Laura M. Herz[4], Xiao Cheng Zeng[2, *], Yuanyuan Zhou [5,11 *]

[1] Department of Applied Physics, The Hong Kong Polytechnic University, Hong Kong SAR, China

[2] Department of Chemistry, University of Nebraska-Lincoln, Lincoln, NE 68588, USA

[3] Qingdao Institute of Bioenergy & Bioprocess Technology, Chinese Academy of Sciences, Qingdao, Shandong 458500, China

[4] Clarendon Laboratory, Department of Physics, University of Oxford, Oxford, OX1 3PU, United Kingdom

[5] Department of Physics, Hong Kong Baptist University, Kowloon, Hong Kong SAR, China

[6] College of Engineering and Applied Sciences and Collaborative Innovation Center of Advanced Microstructures, Nanjing University, Nanjing 210093, China

[7] Department of Physics, University of Warwick, Coventry, CV4 7AL, United Kingdom

[8] Chemistry and Nanoscience Center, National Renewable Energy Laboratory, Golden, Colorado CO, 80401 USA

[9] Material Science Center, National Renewable Energy Laboratory, Golden, Colorado 80401 USA

[10] Renewable and Sustainable Energy Institute and Department of Physics, University of Colorado Boulder, Boulder, Colorado 80309 USA

[11] Smart Society Laboratory, Hong Kong Baptist University, Kowloon, Hong Kong SAR, China

† These authors have contributed equally to this work.

*Correspondence should be addressed to: yyzhou@hkbu.edu.hk; xzeng1@unl.edu; songhua.cai@polyu.edu.hk

This file includes: Supplementary Text; Figures S1 to S22

Supplementary Text

STEM images filtering

Due to the relatively lower dose rate imaging induced signal-to-noise ratio (SNR) degradation than conventional STEM-HAADF acquiring, special processing is needed for further atomic-scale analysis and determination of structural details. Bragg filtering and Butterworth low-pass filtering were applied in this work for STEM-HAADF images processing to improve the SNR, as illustrated in Figures S10, 16. We used 1st order filter for Butterworth filtering. For Bragg filtering, we precisely selected the spots from the fast Fourier transform (FFT) pattern of each image and applied a periodical mask that contains all visible spots. After both of these filter's processing, the SNR of STEM-HAADF images has been improved, especially for Bragg filtering. The structural information of both Butterworth filtered and Bragg filtered images were well retained compared with initial images. Therefore, we chose the Bragg filtered images for further analysis in Figures 2a, 3f and Butterworth filtered image in Figure 3b.

As Bragg filter only reserves structural information which included in the periodical mask, we enlarged the size of circles in the mask to reserve more details, as shown in Figure S10f. For accurate characterization of high angle twin-boundary, we applied two independent Bragg filtering masks corresponding to both of the twisted grains in the same STEM-HAADF image as shown in Figure S16e, f. This helps avoid the potential influence of Bragg filtering to boundary details and keeps all features of the twin-boundary for the reconstruction of atomic structure.

Geometric Phase Analysis (GPA) strain mapping

In this work, GPA strain mapping was used to determine strain distribution resulting from perovskite intragrain heterogeneity (Figure 2g-i). This was done using the FRWRtools plugin for Digital Micrograph (GMS 3), which is based on geometric phase algorithms originally developed by Martin Hÿtch (Ultramicroscopy 1998, 74, 131-146). The g-vectors used for strain analysis correspond to the (002) and (110) spots of orthorhombic $FACsPbI_3$ in the FFT pattern.

Device Characterization and Testing

J-V curves of the as-fabricated PSCs were measured using a 2400 Sourcemeter (Keithley, USA) under simulated one-sun AM 1.5G 100 mW cm^{-2} intensity (Oriel Sol3A Class AAA, Newport, USA). The *J-V* scan rate is 0.1V/s and the dwell time 50 ms. The test was performed in the ambient air. No special preconditioning was applied. Slight hysteresis was observed, which is typical for a perovskite solar cell. No other unusual behavior. The typical active area of PSCs is 0.09 cm^2 defined by a metal mask. The intensity of the one-sun AM 1.5G illumination was calibrated using a Si-reference cell certified by the National Renewable Energy Laboratory.

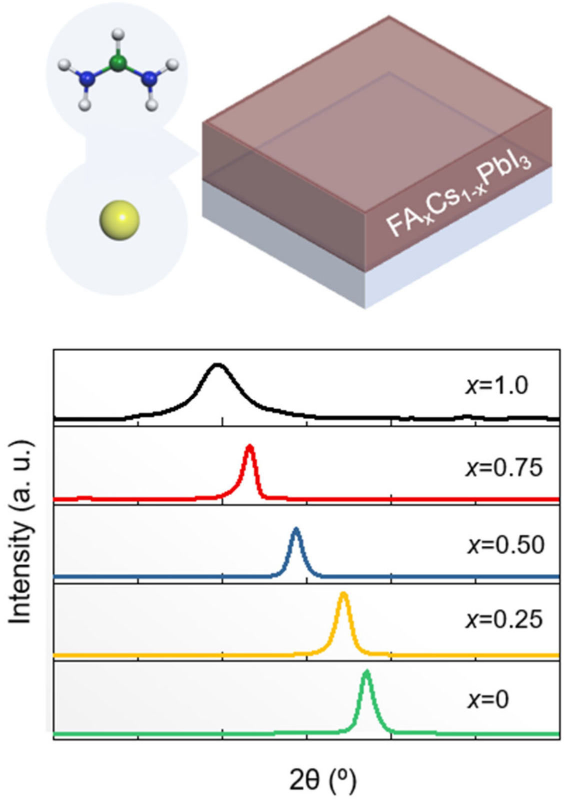

Figure S1. (a) Schematic illustration showing the fabrication of $FA_xCs_{1-x}PbI_3$ films based on FA-Cs cation mixing. (b) XRD patterns of $FA_xCs_{1-x}PbI_3$ perovskite films with different x.

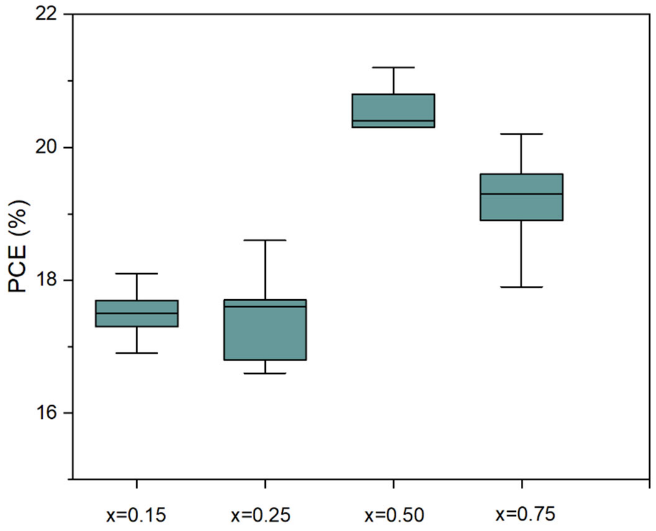

Figure S2. Device PCE statistics of $FA_xCs_{1-x}PbI_3$ perovskite solar cells with different *x*.

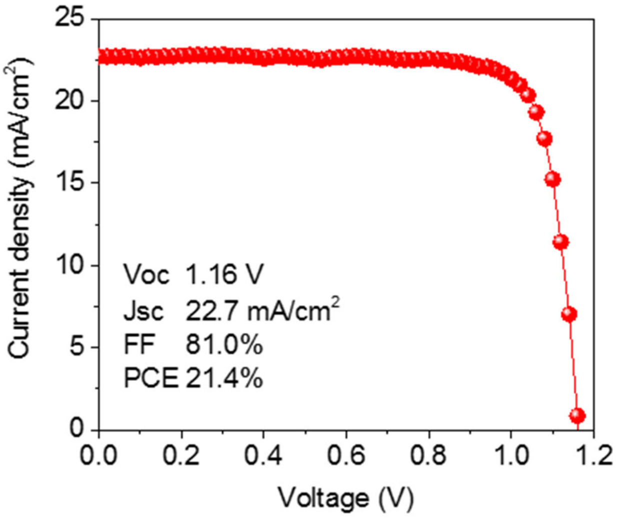

Figure S3. *J*-*V* curve (reverse scan) of the best-performing FA-Cs PSC. The inset shows the extracted *J*-*V* parameters.

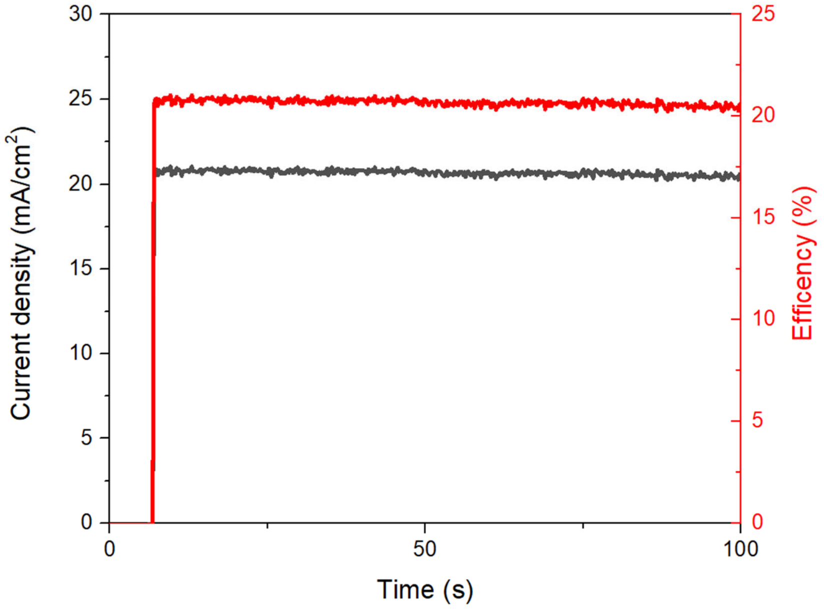

Figure S4. Power and current outputs upon maximum-power-point tracking under one-sun illumination.

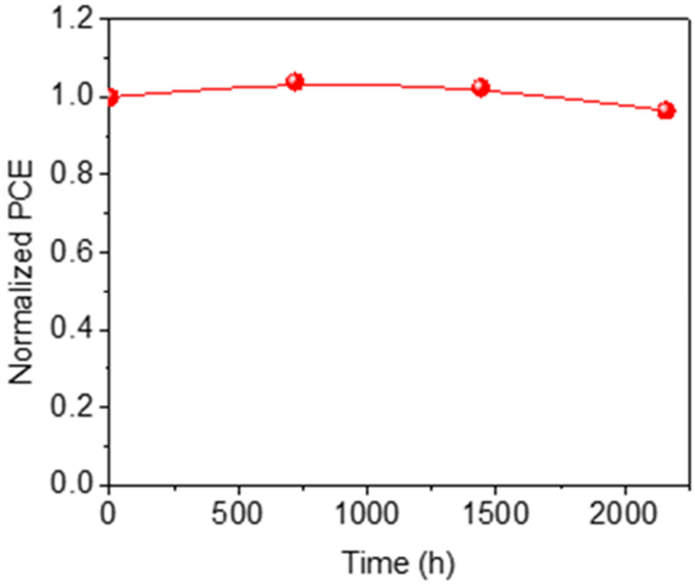

Figure S5. Shelf stability of a $FA_{0.5}Cs_{0.5}PbI_3$ PSC when stored in nitrogen.

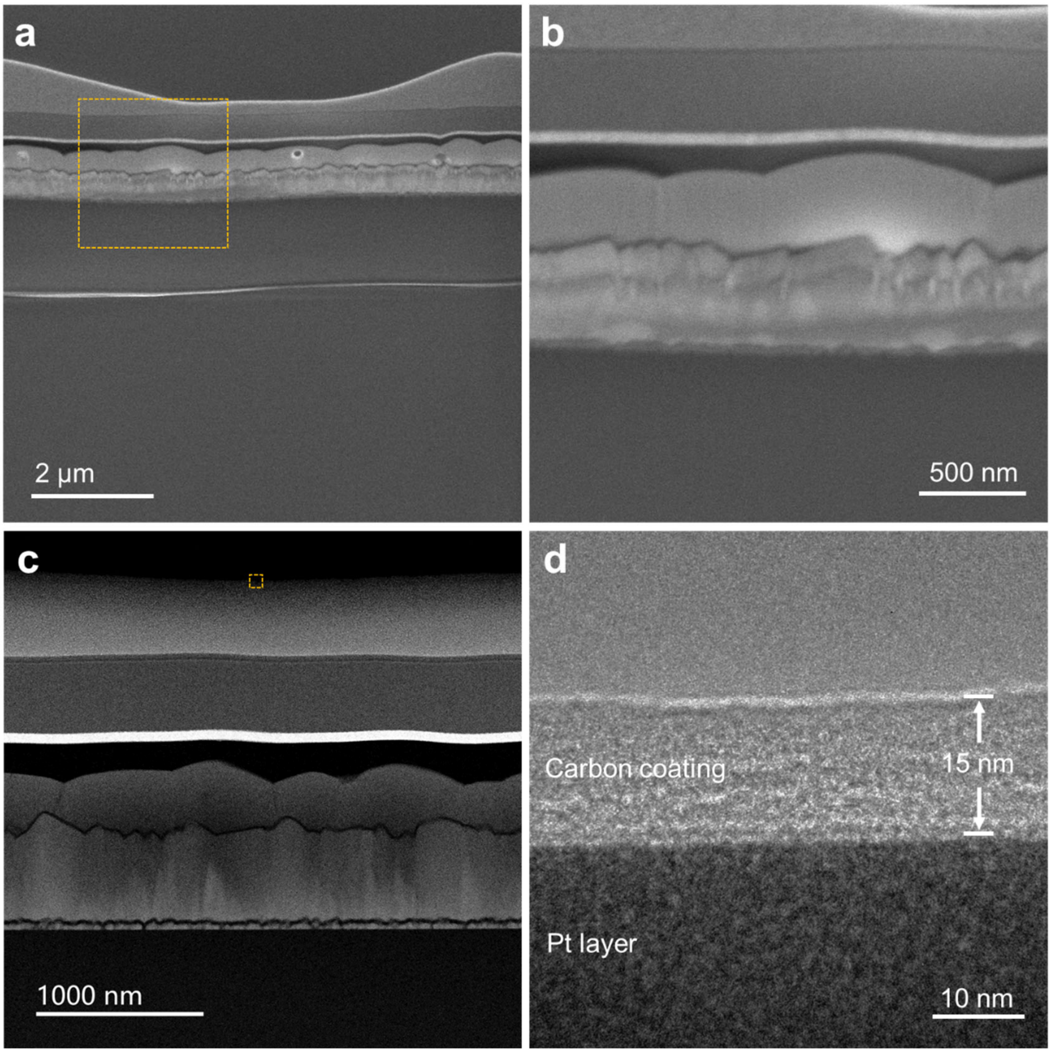

Figure S6. FIB-prepared cross-section specimen of a PSC: (a) Scanning electron microscope (SEM) image of FIB-prepared PSC cross-section specimen. (b) Magnified SEM image taken from yellow squared region in (a). (c) Low-magnification STEM-HAADF image of the PSC cross-section specimen. Note that the Pt layer is deposited during the FIB processing for sample protection. (d) High-magnification TEM image showing the edge of the specimen (corresponding to the labelled region in (a)), revealing the amorphous carbon coating.

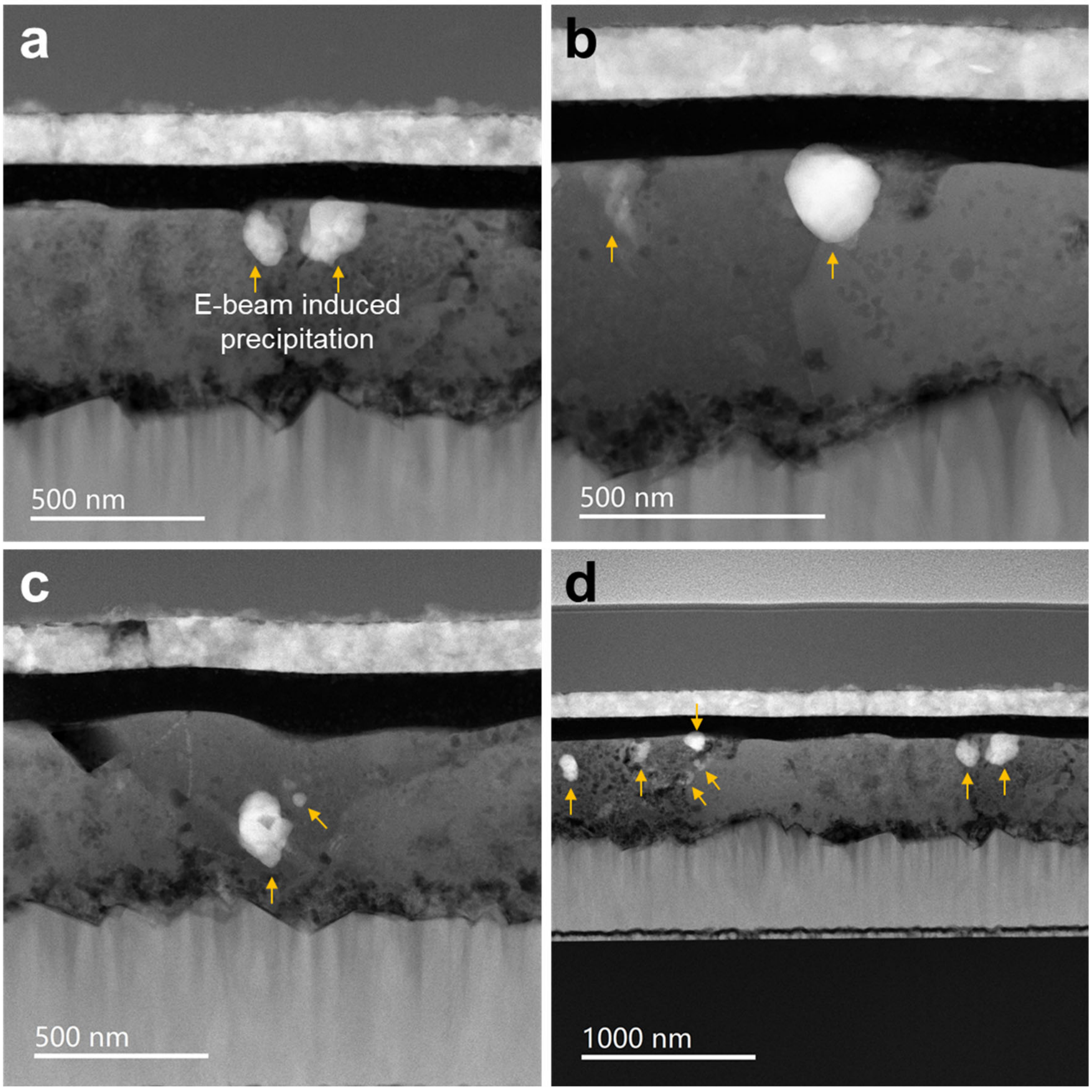

Figure S7. Electron-beam-induced damage on a cross-section specimen without the amorphous carbon coating. Without the carbon coating protection, the FIBed PSC cross-session sample is extremely sensitive to the 300 kV electron beam. Even under the low magnification STEM scanning mode, the electron beam can easily induce the precipitation of heavy atoms which may be mostly formed by Pb as shown in (a-d), resulting in challenges for high magnification STEM characterization.

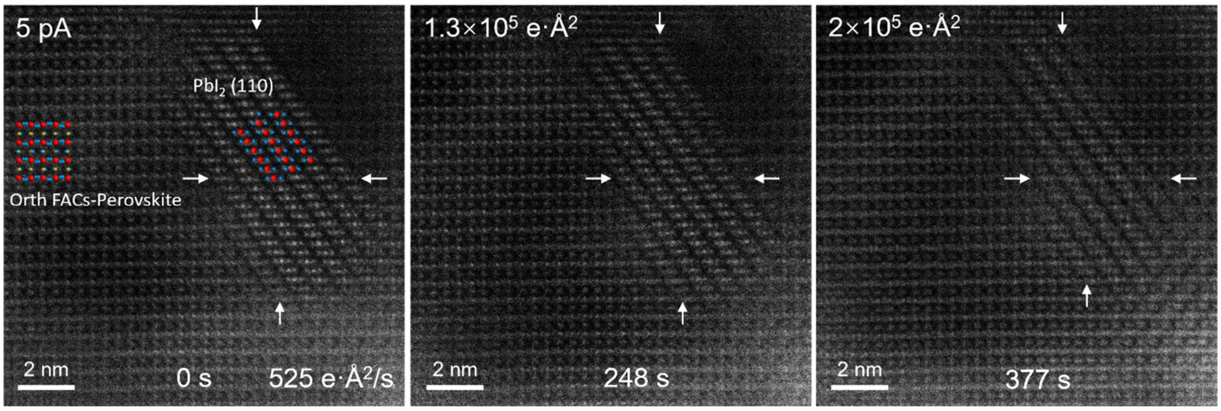

Figure S8. The structural stability of as-prepared cross-section perovskite sample under electron probe scanning. An orthorhombic perovskite region in $FA_{0.5}Cs_{0.5}PbI_3$ grain with PbI_2 cluster was chosen and kept scanning with a 5 pA electron probe ([100] projection direction). Perovskite structure doesn't show obvious change without further degrading to PbI_2 during the continuous 377s electron probe scanning.

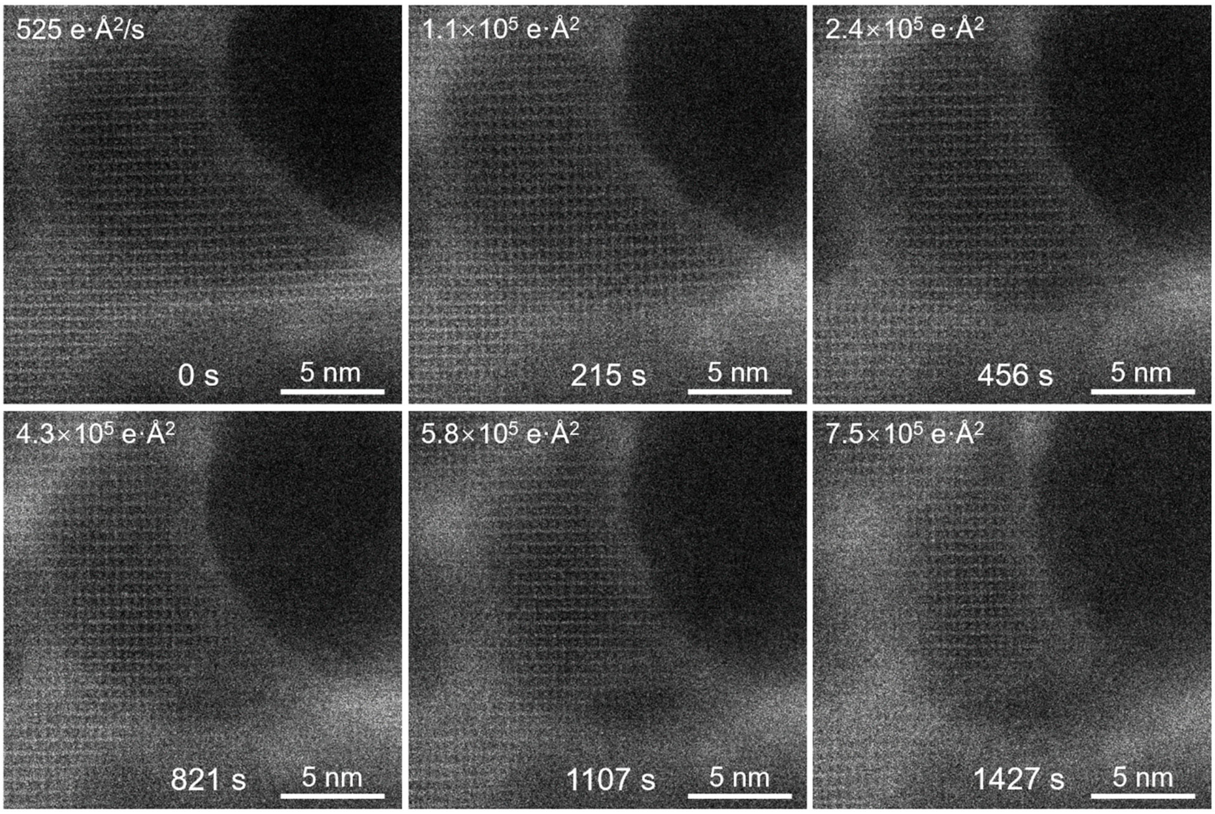

Figure S9. The perovskite structure degradation process under long time electron probe scanning. An orthorhombic perovskite region was chosen and kept scanning with a 5 pA electron probe ([100] projection direction) for 1427 s. As shown in the series STEM-HAADF images, with continuous electron probe scanning, the perovskite structure begins to degrade to an amorphous structure at a very low speed from the surrounding region. During this process, the structure of the remaining perovskite part remains unchanged without degrading to PbI_2. This further confirms the fidelity of low-dose STEM characterization on cross-sectional PSC samples with carbon layer protection.

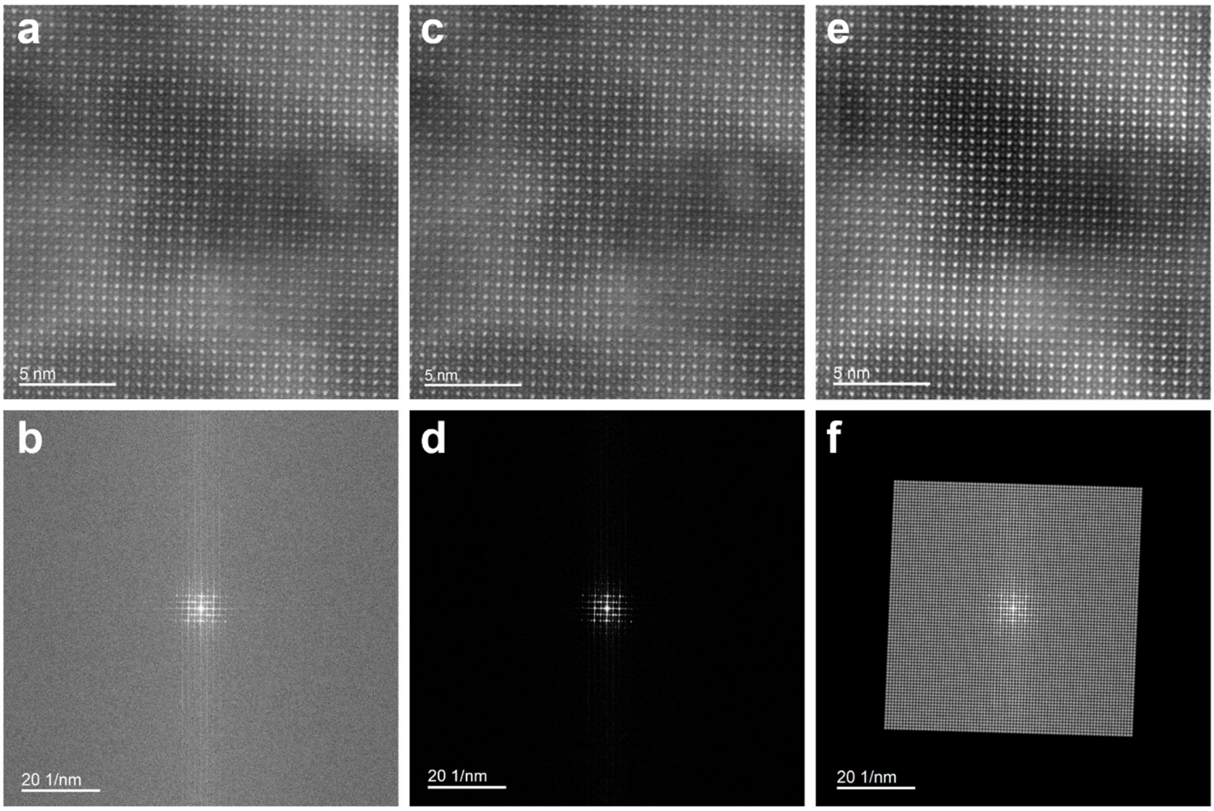

Figure S10. Filtering method for STEM-HAADF images processing: (a) A typical atomic resolution STEM-HAADF image of the orthorhombic FA-Cs perovskite. (b) Corresponding FFT pattern of (a). (c) The same image of (a) after Butterworth low-pass filter was applied. (d) The Fourier transformation of (c). (e) The same image after Bragg filter was applied with a much improved SNR. (f) The periodical mask used for the Bragg filtering in (e).

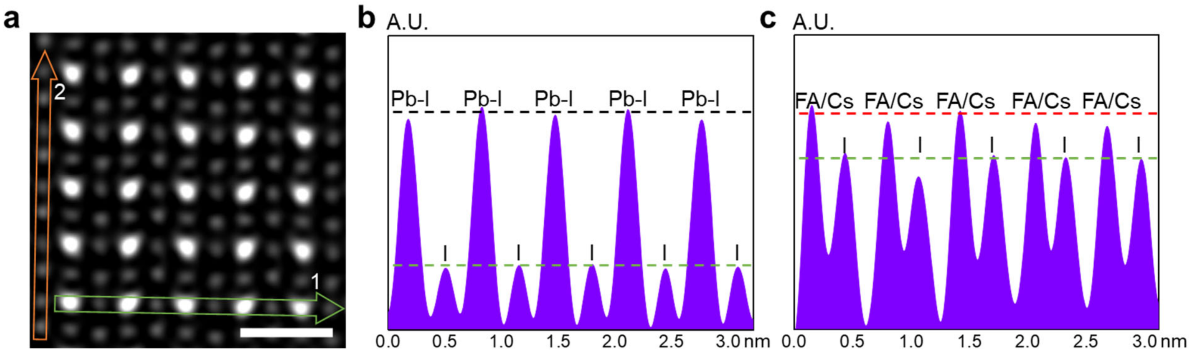

Figure S11. Intensity line profiles of Pb-I and FA/Cs-I atomic columns in atomic-scale STEM-HAADF image: (a) A typical region in Figure 2a. Scale bar: 1 nm. (b) Line profile of Pb-I-I column signal intensity marked by the green arrow in (a), indicating a much higher contrast of Pb-I columns than I columns. (c) Line profile of FA/Cs-I column signal intensity marked by orange arrow in (a), indicating a slightly higher contrast of FA/Cs columns than I columns.

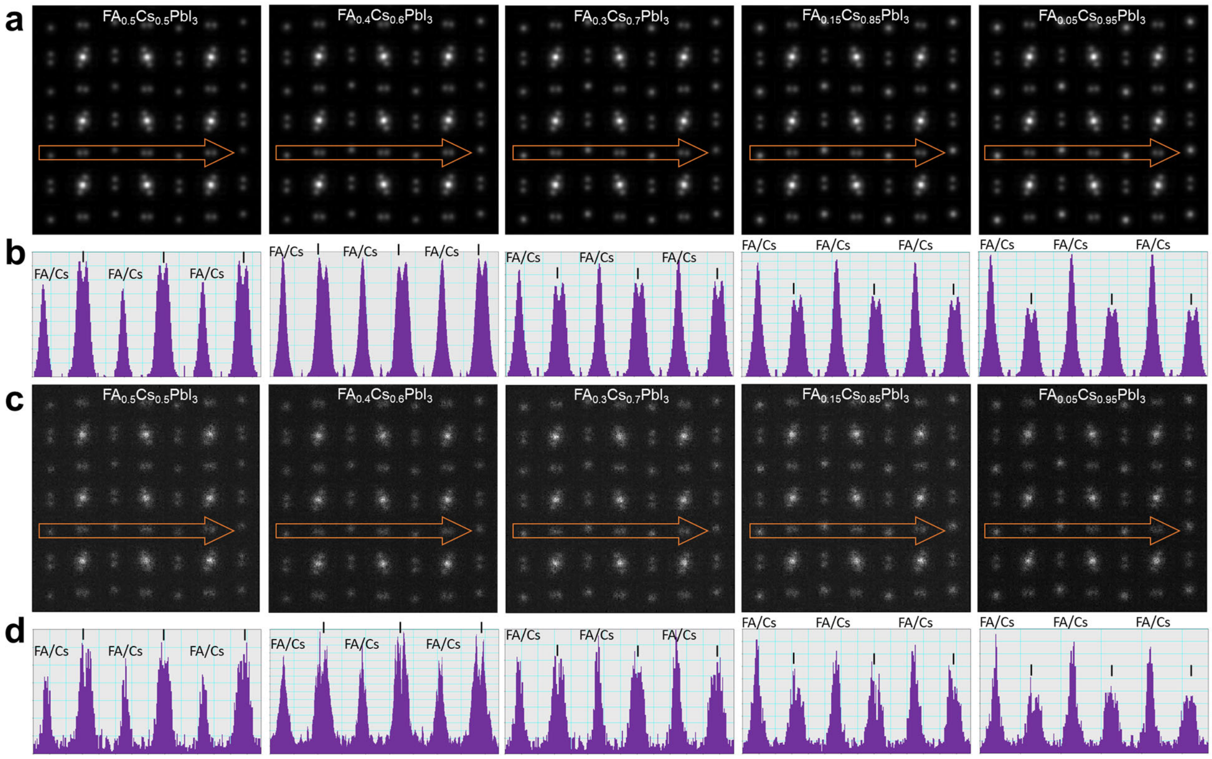

Figure S12. Simulated STEM-HAADF images of FA-Cs perovskites: (a) Qstem simulated pristine STEM-HAADF image of $FA_{0.5}Cs_{0.5}PbI_3$, $FA_{0.4}Cs_{0.6}PbI_3$, $FA_{0.3}Cs_{0.7}PbI_3$, $FA_{0.15}Cs_{0.85}PbI_3$, $FA_{0.05}Cs_{0.95}PbI_3$ from $[1\bar{1}0]$ projection direction, respectively. The accelerating voltage of electron probe is 300 kV, convergence angle is 22.5 mrad, acquisition angle is 79.5 mrad to 200 mrad, similar with experiment conditions. (b) Corresponding line profiles of FA/Cs-I column signal intensity marked by orange arrow in (a). (c) Simulated STEM-HAADF images similar with (a) after Poisson noise processing, makes them closer to experimental result. (d) Corresponding line profiles of FA/Cs-I column signal intensity marked by orange arrow in (c).

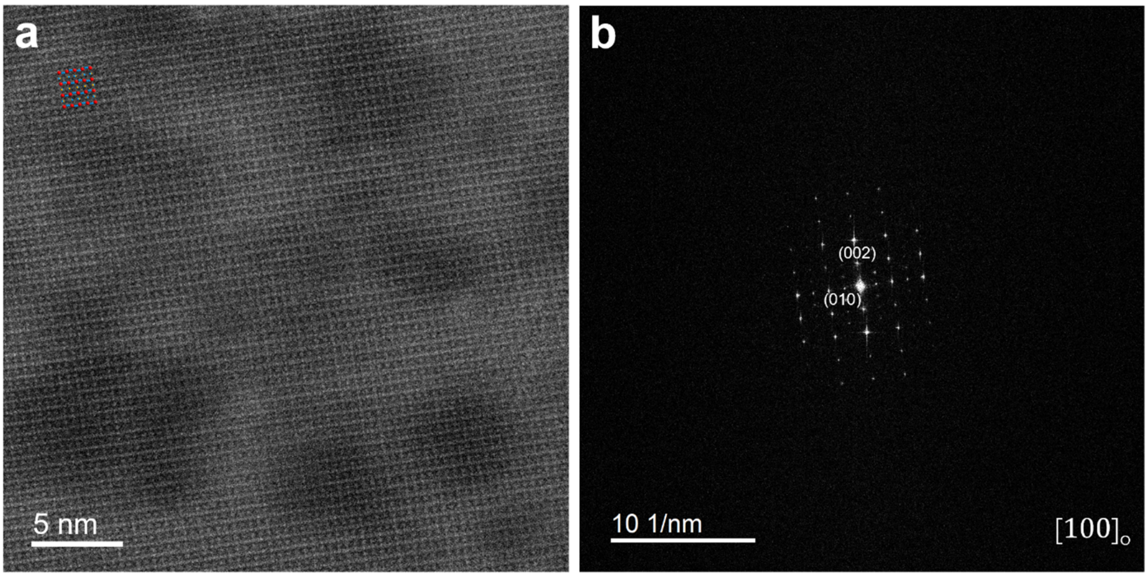

Figure S13. Orthorhombic FA-Cs perovskite along $[100]_o$ projection direction: (a) Atomic resolution unfiltered STEM-HAADF image of $FA_{0.5}Cs_{0.5}PbI_3$ along $[100]_o$ projection direction, consistent with corresponding atom structure model. The uneven contrast results from the ion beam etching nonuniformity in FIB sample preparation. (b) FFT pattern of (a).

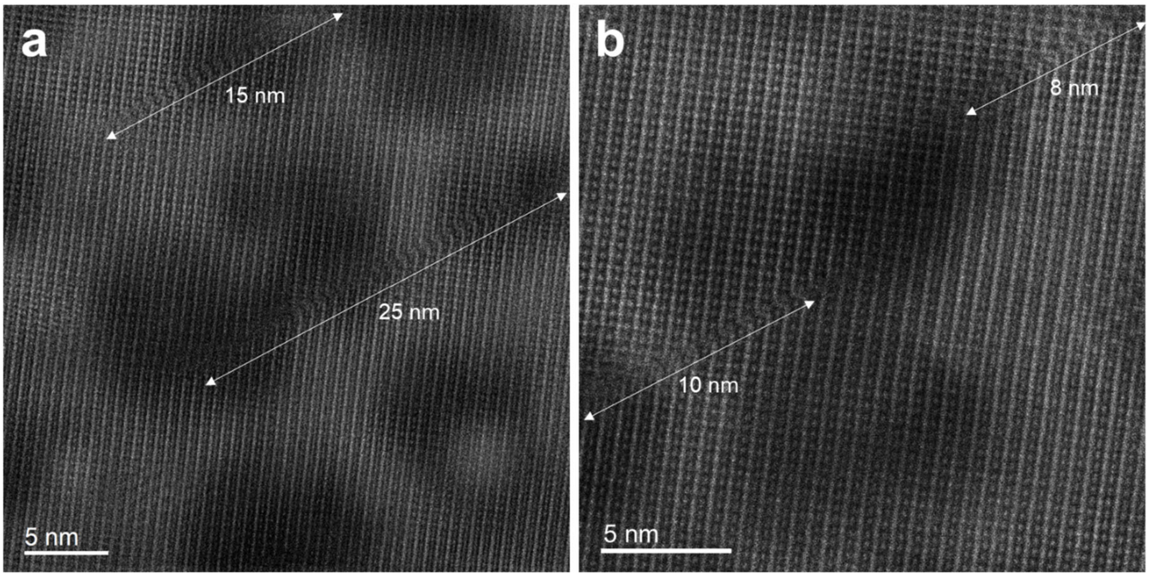

Figure S14. Stacking faults in orthorhombic FA-Cs perovskite: (a, b) STEM-HAADF images of stacking faults in $FA_{0.5}Cs_{0.5}PbI_3$ along $[100]_o$ projection direction, exhibit a characteristic length of about 8-25 nm.

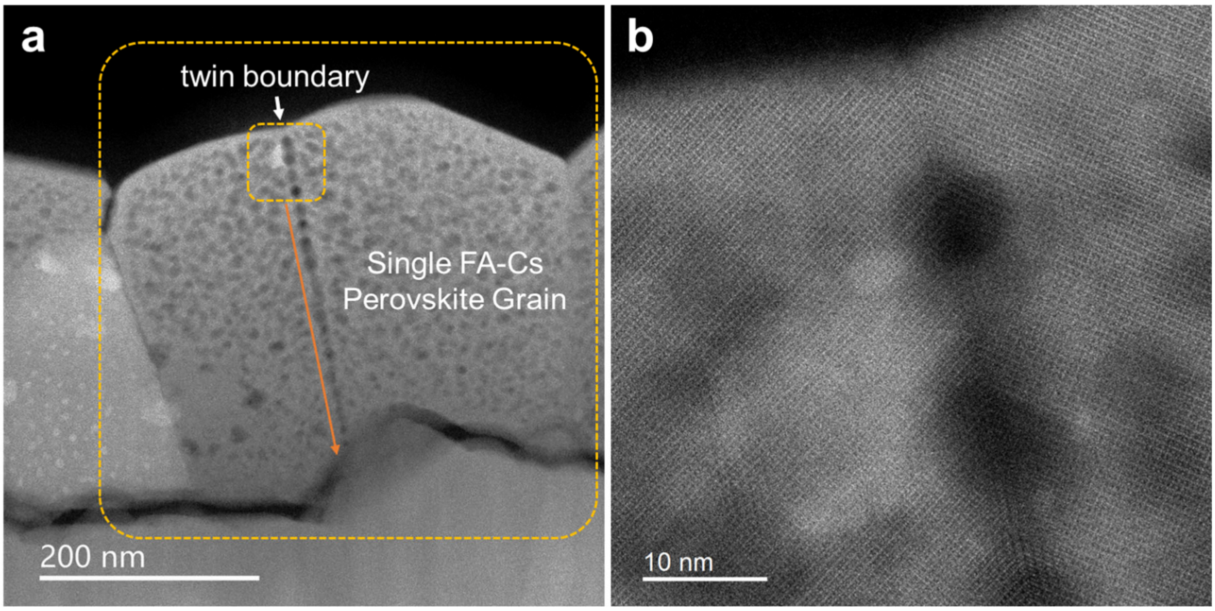

Figure S15. Twin-boundary throughout the film thickness in orthorhombic FA-Cs perovskite: (a) Low magnification STEM-HAADF image of FA-Cs perovskite thin films with twin-boundary across the film thickness. (b) High magnification unfiltered STEM-HAADF image of the twin-boundary region marked by yellow square in (a).

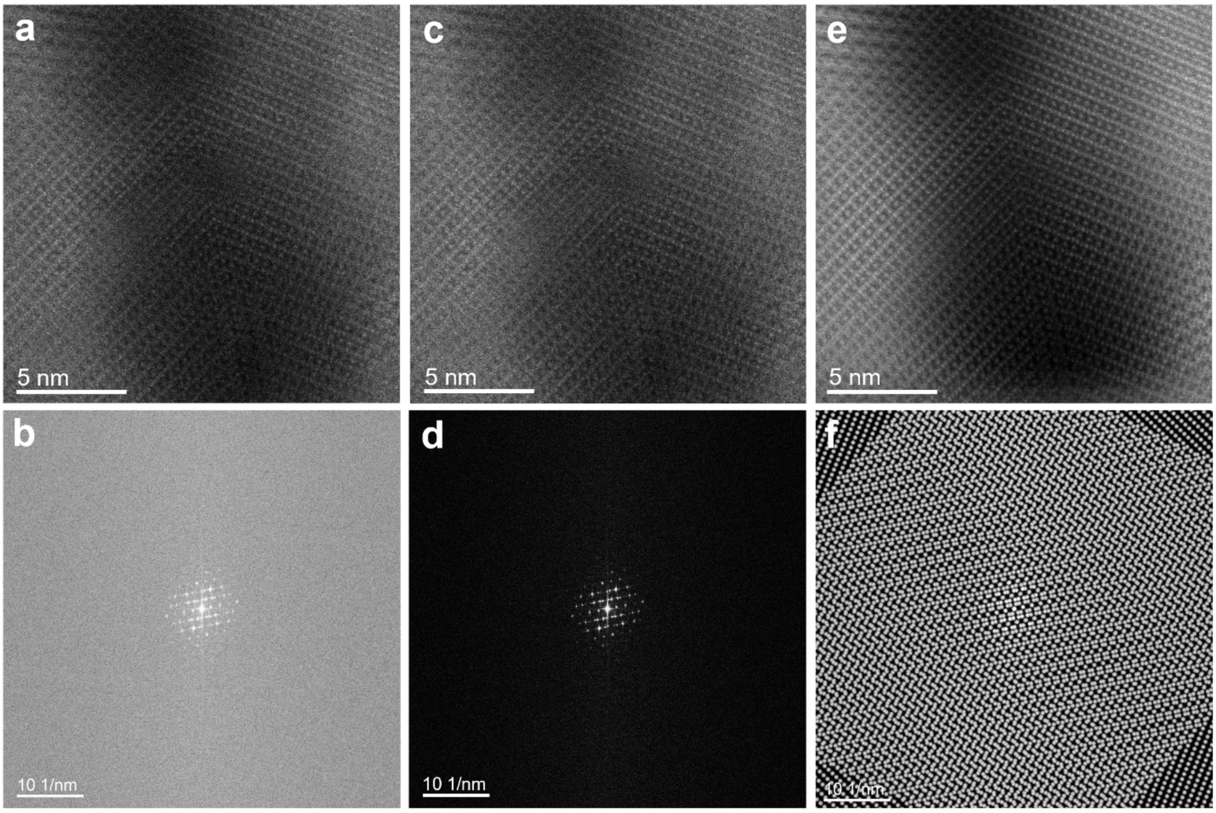

Figure S16. Filtering method for processing of twin-boundary images: (a) A typical atomic resolution STEM-HAADF image of high angle twin-boundary. (b) Corresponding FFT pattern of (a). (c) The same image of (a) after Butterworth low-pass filter was applied. (d) The Fourier transformation of (c). (e) The same image of (a) after Bragg filter was applied with a better SNR. (f) Two independent periodical masks fit for both of the twisted grains used for Bragg filtering in (e).

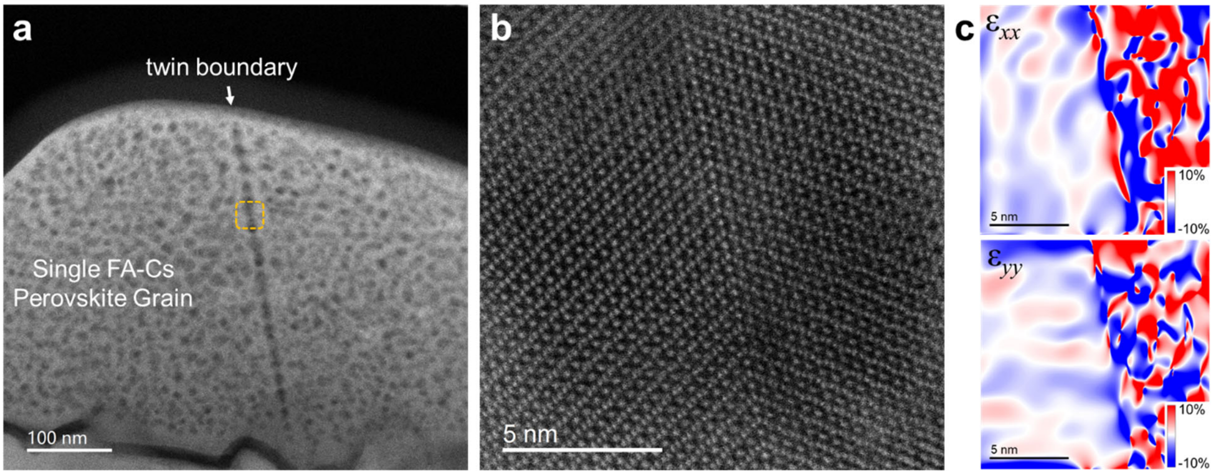

Figure S17. The existence of representative twin-boundary in FA-Cs PSCs: (a) A twin-boundary observed in another $FA_{0.5}Cs_{0.5}PbI_3$ grain similar with the case in Figure 3. (b) Butterworth filtered atomic-scale STEM-ADF image confirms the same structure of this representative twin-boundary. It is worth noting that ADF image can provide a better SNR than HAADF image with a higher acquiring fraction of electrons, and both the HAADF images in Figures 3f, S15 and ADF image in (b) reveals a similar atomic structure. (c) In-plane strain ε_{xx} and ε_{yy} distribution of (b) generated by GPA analysis, showing a dramatic lattice distortion at twin boundary. As this twin-boundary is a high-angle twin-boundary, and the GPA analysis uses the left domain for reference, therefore leading to the artifacts in right domain.

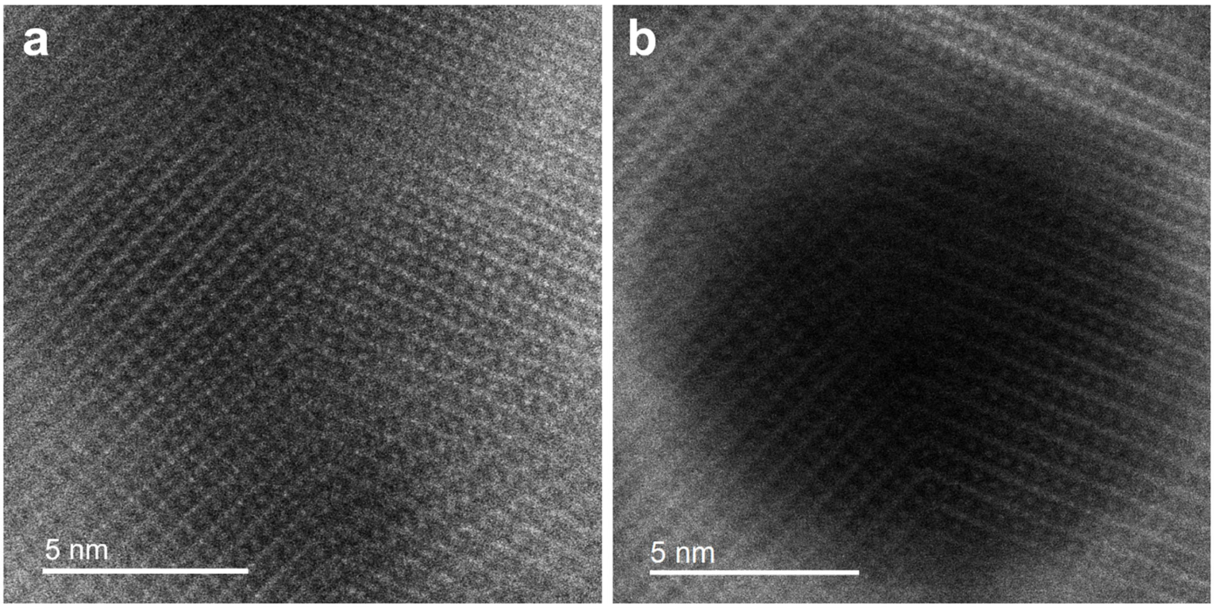

Figure S18. The electron probe induced structure damage on twin-boundary: (a) Atomic-scale unfiltered STEM-HAADF image taken from a twin-boundary region in $FA_{0.5}Cs_{0.5}PbI_3$ grain similar with the case in Figure 3, reveals the initial structure. (b) After one-minute electron probe scanning, an obvious dark region occurs in the core of the twin-boundary, which implies the electron probe induced damage. However, the remaining part still retains the perovskite structure without phase transition or degradation, also the twin-boundary structure remains unchanged compared with (a) and Figures 3f, S15, 16. This further confirms the fidelity of STEM characterized perovskite twin-boundary structure.

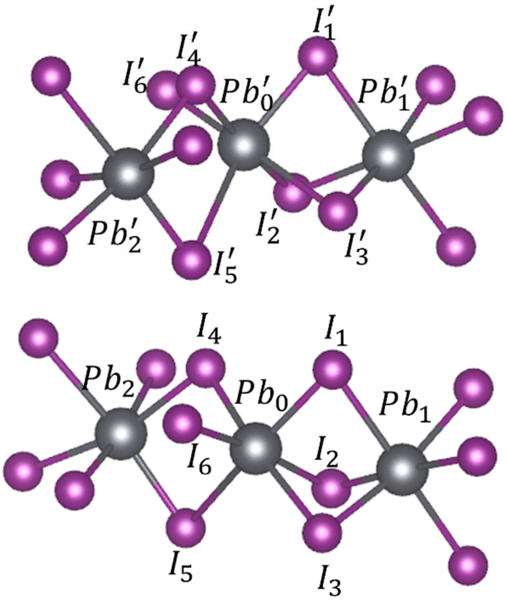

$Pb_0' - I_1'$: 3.045 Å
$Pb_0' - I_2'$: 3.314 Å
$Pb_0' - I_3'$: 3.333 Å
$Pb_0' - I_4'$: 3.160 Å
$Pb_0' - I_5'$: 4.299 Å
$Pb_0' - I_6'$: 3.190 Å
$Pb_0' - Pb_1'$: 3.948 Å
$Pb_0' - Pb_2'$: 4.908 Å

$I_1' - Pb_0' - I_3'$: 88.001°
$I_1' - Pb_0' - I_2'$: 88.312°
$I_2' - Pb_0' - I_3'$: 82.007°
$Pb_1' - Pb_0' - Pb_2'$:141.959°

Figure S19. The optimized local atomic structure of the stacking fault interface. An initial structure of the stacking fault was created by a shift along the (011) plane by 0.2 unit, followed by relocating two Pb atoms (Pb_0 and Pb_0') to resemble the experimental interfacial structure (Figure 3b, c). After structural optimization, a reconstruction of local Pb-I bonding pattern occurs in central part of the interface. Specifically, the Pb and its nearest-neighboring Pb distance (e.g., Pb_0 - Pb_1 and Pb_0' - Pb_1') is notably shortened compared to those (> 6 Å) in the bulk crystal. As a result, three iodine atoms (I_1', I_2', I_3') form three bent structures with Pb_0' and Pb_1', namely, $Pb_0' - I_1' - Pb_1'$, $Pb_0' - I_2' - Pb_1'$, and $Pb_0' - I_3' - Pb_1'$ bent structures. Here, Pb_0' - I_1' has the shortest Pb-I bond in the upper part (likewise, Pb_0 - I_1 has the shortest Pb-I bond in the lower part). After an I vacancy at the location of I_1 is created (i.e., the location marked by a red dashed circle in Figure 5d), the optimized stacking fault structure gives rise to deep trap states as shown in the middle and lower panels of Figure 5d.

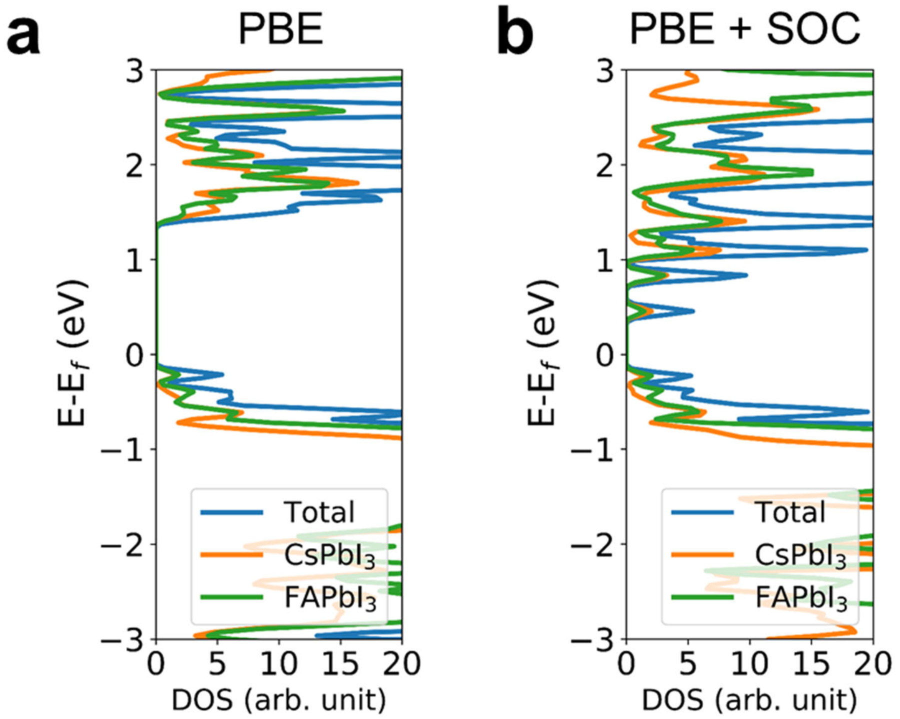

Figure S20. The total DOS and partial DOS of the left ($CsPbI_3$) and right ($FAPbI_3$) domains of the composition-boundary interface based on different DFT calculations: (a) PBE calculation result similar with that shown in Figure 4a. (b) PBE+SOC (spin-orbit coupling) calculation result.

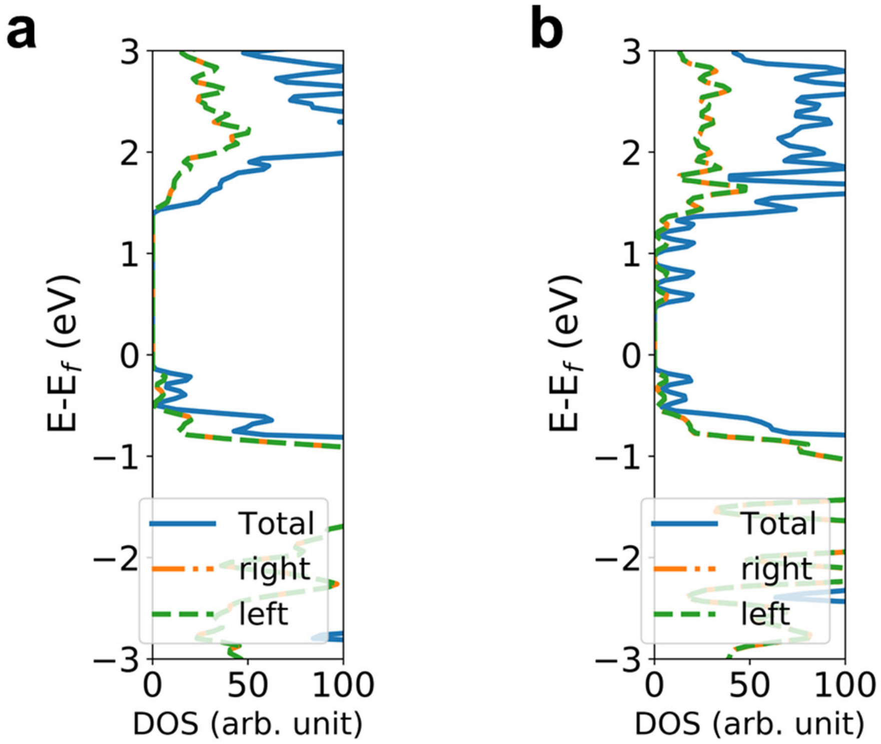

Figure S21. The total DOS and partial DOS of the left and right domains of the twin-boundary interface based on different DFT calculations: (a) PBE calculation result similar with Figure 4c. (b) PBE+SOC (spin-orbit coupling) calculation result.

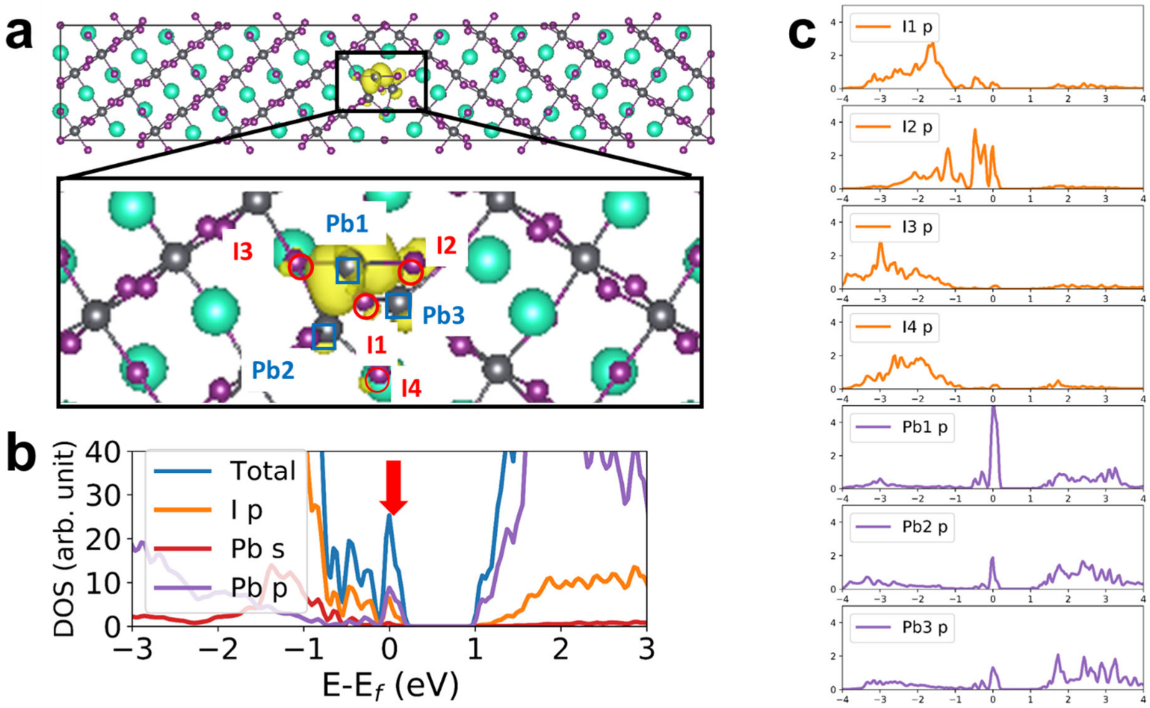

Figure S22. The charge density corresponding to the VBM, the total DOS (TDOS) and partial DOS (pDOS) of selected atoms in the twin-boundary interface with a Pb interstitial: (a) Charge density (yellow area) of the shoulder peak near the band edges of the twin-boundary interface with one Pb interstitial. (b) Total DOS, and partial DOS for Pb *s, p* orbitals and I *p* orbitals. The VBM exhibits localized trap states. (c) The selected Pb and I atom shown in the pDOS (bottom panel) is marked with blue square and red circles in the structure shown in (a).
